# LogBTF: gene regulatory network inference using Boolean threshold network model from single-cell gene expression data

**DOI:** 10.1093/bioinformatics/btad256

**Published:** 2023-04-20

**Authors:** Lingyu Li, Liangjie Sun, Guangyi Chen, Chi-Wing Wong, Wai-Ki Ching, Zhi-Ping Liu

**Affiliations:** Department of Biomedical Engineering, School of Control Science and Engineering, Shandong University, Jinan 250061, China; Advanced Modeling and Applied Computing Laboratory, Department of Mathematics, The University of Hong Kong, Hong Kong, China; Advanced Modeling and Applied Computing Laboratory, Department of Mathematics, The University of Hong Kong, Hong Kong, China; Department of Biomedical Engineering, School of Control Science and Engineering, Shandong University, Jinan 250061, China; Advanced Modeling and Applied Computing Laboratory, Department of Mathematics, The University of Hong Kong, Hong Kong, China; Advanced Modeling and Applied Computing Laboratory, Department of Mathematics, The University of Hong Kong, Hong Kong, China; Department of Biomedical Engineering, School of Control Science and Engineering, Shandong University, Jinan 250061, China

## Abstract

**Motivation:**

From a systematic perspective, it is crucial to infer and analyze gene regulatory network (GRN) from high-throughput single-cell RNA sequencing data. However, most existing GRN inference methods mainly focus on the network topology, only few of them consider how to explicitly describe the updated logic rules of regulation in GRNs to obtain their dynamics. Moreover, some inference methods also fail to deal with the over-fitting problem caused by the noise in time series data.

**Results:**

In this article, we propose a novel embedded Boolean threshold network method called LogBTF, which effectively infers GRN by integrating regularized logistic regression and Boolean threshold function. First, the continuous gene expression values are converted into Boolean values and the elastic net regression model is adopted to fit the binarized time series data. Then, the estimated regression coefficients are applied to represent the unknown Boolean threshold function of the candidate Boolean threshold network as the dynamical equations. To overcome the multi-collinearity and over-fitting problems, a new and effective approach is designed to optimize the network topology by adding a perturbation design matrix to the input data and thereafter setting sufficiently small elements of the output coefficient vector to zeros. In addition, the cross-validation procedure is implemented into the Boolean threshold network model framework to strengthen the inference capability. Finally, extensive experiments on one simulated Boolean value dataset, dozens of simulation datasets, and three real single-cell RNA sequencing datasets demonstrate that the LogBTF method can infer GRNs from time series data more accurately than some other alternative methods for GRN inference.

**Availability and implementation:**

The source data and code are available at https://github.com/zpliulab/LogBTF.

## 1 Introduction

With the tremendous progress of advanced technology and the improvement of sensitivity of cell analysis, single-cell RNA sequencing (scRNA-seq) data have brought unprecedented challenges and opportunities for deciphering the regulatory relationship among genes (Chen and Liu 2022; Luo et al. 2022). One challenge of scRNA-seq data analytics is its “dropout” (Qiu 2020). Because of the dropouts, scRNA-seq data are extremely sparse (excessive zero counts) so as to only capture a little information about the transcriptome of each cell (Qiu 2020). Despite this, the analysis for scRNA-seq data promotes the development of gene regulatory network (GRN) inference methods to help us explore the mechanisms underlying various biological processes (Papili Gao et al. 2018; Algabri et al. 2022) and expectedly develop efficient therapies to treat and cure diseases (Aalto et al. 2020). Recently, GRN inference from time series data has gained more and more attention (Aalto et al. 2020). In particular, the studies of scRNA-seq provide an ordering of cells involved in a dynamical process (such as differentiation) through inferred pseudo-time associated with each cell (Zhang et al. 2021). Furthermore, the pseudo-time can be regarded as temporal information for single-cell gene expression profiles, which offers a unique opportunity to infer GRNs (Aubin-Frankowski and Vert 2020).

From the systems biology point of view, inferring GRN plays an extremely crucial role in revealing underlying regulatory mechanisms to uncover potential genes (Akutsu et al. 2000; Zhang et al. 2012; Liu et al. 2021). A vast number of network inference/reconstruction methods have been widely developed to infer GRN using transcriptomic profiles. To the best of our knowledge, the existing network inference/reconstruction methods can be categorized into the following groups according to their principles (Liu 2015): regression-based method [multiple regression model (Zhang et al. 2010), SINCERITIES (Papili Gao et al. 2018), and GNIPLR (Zhang et al. 2021)], tree-based method [GENIE3 (Huynh-Thu et al. 2010)], stability selection method [TIGRESS (Haury et al. 2012)], correlation-based method [ARACNE (Margolin et al. 2006) and CLR (Faith et al. 2007)], knowledge-based method [RegNetwork (Liu et al. 2015)], ordinary differential equation method [linear ODE (Wu et al. 2014), SCODE (Matsumoto et al. 2017), and GRISLI (Aubin-Frankowski and Vert 2020)], Bayesian-based method [SSMs (Beal et al. 2005), Vireo (Huang et al. 2019), and NAE (Wang et al. 2022)], Boolean network (BN) model method [ATEN (Shi et al. 2020) and GAPORE (Liu et al. 2021)], and deep learning model [DeepDRIM (Chen et al. 2021), dynDeepDRIM (Xu et al. 2022), and DeepSEM (Shu et al. 2021)].

Generally speaking, there are four levels of inferring GRNs (Liu 2015). (i) Is there a regulatory relationship? (ii) Who is the regulator, and who is the target? (iii) Whether the regulatory relationship is activating or inhibiting? (iv) How strong is this regulatory relationship? Some GRN reconstruction methods (e.g. gene co-expression network) can infer a network with undirected edges, which only reflect the associations among genes and build the fundamental architecture of these regulations, i.e. at the level i. Some methods can identify the regulatory direction (level ii) but cannot obtain extra regulatory information, such as regulatory mechanism (level iii), and relative regulatory strength (level iv).

Several data-driven methods have been applied to infer GRNs (Luo et al. 2022). For example, the accurate cellular network reconstruction algorithm (ARACNE) method assumes that correlation analysis can be used to discover regulatory interactions between genes by considering a time gap between gene expression values (Margolin et al. 2006). However, such statistical dependence between the studied genes does not necessarily capture the regulatory relationships (Aubin-Frankowski and Vert 2020). The context likelihood of relatedness (CLR) method reduces the connections from a complete graph by discarding false connections through comparisons of pairwise mutual information scores with a background correction of mutual information scores (Faith et al. 2007). The CLR method effectively saves computational costs, but it only evaluates pairwise interactions (Barman and Kwon 2017). Later, by performing feature selection problems, several ensemble methods are proposed to provide a set of solutions rather than a single solution. For example, the gene network inference with the ensemble of trees (GENIE3) method selects a set of features for extra trees or random forests learning models (Huynh-Thu et al. 2010), and it is regarded as an effective algorithm for reconstructing GRN on both scRNA-seq and bulk RNA-seq gene expression data (Chen et al. 2021). The trustful inference of gene regulation using stability selection (TIGRESS) method conducts feature selection using the least angle regression integrated with a stability selection (Haury et al. 2012). Although these two methods show satisfactory performances, they are only intended to infer the regulatory structure without considering regulatory rules or functions. Also, the inferred GRN may include many false positives and indirect regulations since they are only based on relevance (Aibar et al. 2017). What’s more, single-cell regulatory network inference and clustering (SCENIC) is a typical computational method to infer GRNs and cell types from scRNA-seq data (Aibar et al. 2017). However, it still establishes all possible regulatory relationships using GENIE3 in its first step. Therefore, ones have to develop a novel approach to infer the regulatory rules for the prediction of network dynamics.

The BN was first introduced by Kauffman (1969) to qualitatively describe gene regulatory interactions to account for a variety of complex biological processes. Over the past few decades, BNs have been attracting the great attention of many researchers (Akutsu et al. 1999; Sun et al. 2021; Mori and Akutsu 2022; Sun and Ching 2023). BNs are not only a fundamental model for genetic systems that identify network structures from a systematic perspective (Liu 2015), but also a powerful framework for studying and modeling the dynamics of GRNs (Shi et al. 2020). In the face of tens of thousands of such high-dimensional gene expression time series data, traditional ordinary differential equation models face very intractable difficulties in solving and inferring the network topology, especially sufficient storage space and expensive computing cost. In contrast, BNs faithfully reproduce the states obtained with more realistic continuum reaction kinetics models by simplifying regulatory interactions between genes using discrete variables (Font-Clos et al. 2021). Furthermore, although the major obstacle in inferring GRNs is the dropouts of data (Aalto et al. 2020), Qiu (2020) has illustrated that the dropout pattern in scRNA-seq data is an extremely useful signal by binarizing the count matrix, i.e. setting all non-zero observations into 1 and all dropouts are still 0, and pointed out that recognizing the utility of dropouts suggests an alternative direction for developing computational algorithms for scRNA-seq data.

In this article, we demonstrate an application of the Boolean threshold network model conceived to integrate time series single-cell data into a **Log**istic regression estimation-based **B**oolean **T**hreshold **F**unction (called LogBTF method) to infer GRN by synchronous evolution. First, LogBTF embeds the coefficients estimated by regularized logistic regression into the Boolean threshold network model, which perfectly controls the in-degree of nodes and addresses the problem of over-fitting. Second, LogBTF employs the knowledge of the perturbation design to optimize the inferred network topology, which successfully handles the multi-collinearity problem caused by binarized gene expression data. Third, LogBTF is a kind of interpretable network inference method that could comprehensively output more detailed information about regulatory relationships, such as regulator or target, activation or inhibition, and relative regulatory strength. Moreover, numerous experiments conducted on artificial Boolean datasets, simulated single-cell datasets, and real scRNA-seq datasets demonstrate the effectiveness and efficiency of the LogBTF method. Lastly, the comparison study with eight existing GRN inference methods shows that the LogBTF method unearths potential regulatory relationships and obtains better inference performances simultaneously.

## 2 Materials and methods

### 2.1 Boolean threshold network model

#### 2.1.1 Boolean threshold network

A BN model works on a directed graph network. It consists of a set of nodes representing the elements of a system, and the state of each node is quantified as 0 (false/not expressed) or 1 (true/expressed). At each discrete time, the state of each node is updated by the state of its neighbor (the node pointing to it) at the previous moment through a rule called Boolean function. Therefore, the edges in the BN represent the regulatory relationships between elements. Generally speaking, the Boolean function is expressed as a statement that acts on the inputs through a logical function using logical operators NOT, AND, OR, etc.; clearly, this statement also returns a False/True state. Hence, suppose *N* is the total number of genes, the updating scheme of $x_{i},\;i\in [1,N]$ can be described as follows: where *f_i_* is a Boolean function, $x_{i}(t+1)$ is the state of the target node *x_i_* at the time point *t *+* *1, and $x_{i_{1}}(t),x_{i_{2}}(t),\ldots ,x_{i_{k_{i}}}(t)$ are the states of the regulatory nodes $x_{i_{1}},x_{i_{2}},\ldots ,x_{i_{k_{i}}}$ at the time point *t*. Here, $i_{1},i_{2},\ldots ,i_{k_{i}}\in [1,N]$ and *k_i_* is the in-degree of the node *x_i_*, denoting the number of regulatory nodes of the target node *x_i_*.


(1) $$x_{i}(t+1)=f_{i}(x_{i_{1}}(t),x_{i_{2}}(t),\ldots ,x_{i_{k_{i}}}(t)),$$

BNs with threshold functions are called Boolean threshold networks, which is a special subset of BNs (Melkman et al. 2018; Cheng et al. 2021). Here the threshold function is a special kind of Boolean function, which can be calculated by a linear threshold unit. It is defined as follows (Anthony 2001):

Definition 2.1. *Let N Boolean inputs be* $x_{1},x_{2},\ldots ,x_{N}$*. A threshold function f on* $x_{1},x_{2},\ldots ,x_{N}$*has the form:where l_i_ is either x_i_ or* $\bar{x_{i}}$*for* $i\in [1,N],\;(w_{1},w_{2},\ldots ,w_{N})\in R^{N}$*is the weight-vector, and* θ∈R*is the threshold.*

(2) $$f(x_{1},x_{2},\ldots ,x_{N})=\{\begin{array}{cc} 1, & w_{1}l_{1}+w_{2}l_{2}+\cdots +w_{N}l_{N}\geq \theta ,\\ 0, & \text{otherwise}, \end{array}$$

Compared with BNs, Boolean threshold networks can easily be implemented and are very suitable for representing regulatory networks (Bornholdt 2008; Zañudo et al. 2011). Here, Boolean threshold network model is employed to infer the complex dynamics of GRN. The updating scheme of $x_{i},\;i\in [1,N]$ can be rewritten as follows:


(3) $$\begin{array}{c} x_{i}(t+1)=f_{i}(x_{i_{1}}(t),x_{i_{2}}(t),\ldots ,x_{i_{k_{i}}}(t))\\ =\{\begin{array}{cc} 1, & w_{i_{1}}l_{i_{1}}+w_{i_{2}}l_{i_{2}}+\cdots +w_{i_{k_{i}}}l_{i_{k_{i}}}\geq \theta_{i},\\ 0, & \text{otherwise}. \end{array} \end{array}$$

For each $l_{j},\;j=i_{1},i_{2},\ldots ,i_{k_{i}}$, if *l_j_* = *x_j_*, the regulatory node *x_j_* promotes the target node *x_i_*; if $l_{j}=\bar{x_{j}}$, the regulatory node *x_j_* inhibits the target node *x_i_*.

#### 2.1.2 Logistic regression model

The logistic regression model is applied to estimate parameters of the above Boolean threshold network model using time series gene expression data. Let *T* be the total number of time points. For each node $x_{i},\;i\in [1,N]$, there are *T−*1 observations $\left(X^{i}(j),y^{i}(j)),\;i\in [1,N],j\in [1,T-1\right]$, which are independent and identical distributed. Let $D_{i}=\{(X^{i}(1),y^{i}(1)),(X^{i}(2),y^{i}(2)),\ldots ,(X^{i}(T-1),y^{i}(T-1))\}$ where $X^{i}(j)={\left(x_{1}(j),x_{2}(j),\ldots ,x_{N}(j),1\right)}^{⊤}\in R^{N+1},\;x_{1}(j),x_{2}(j),\ldots ,x_{N}(j)$ represent the states of nodes (genes) in the *j*-th observation (or at the time point *j*), j∈[1,T−1], and $y^{i}(j)$ is the state of the node *x_i_* at the time point *j *+* *1, and the value is either 0 or 1, i.e. $y^{i}(j)=x_{i}(j+1)$.

The logistic regression is considered as follows: where $\theta_{i}={\left(\theta_{1}^{i},\theta_{2}^{i},\ldots ,\theta_{N}^{i},\theta_{0}^{i}\right)}^{⊤}$, $\theta_{k}^{i}\;(k\in [1,N])$ are the unknown coefficients to be estimated, and $\theta_{0}^{i}$ is the intercept to be estimated, f(·) is the logistic function used to predict *y* for any input of class labels (0 or 1) (James et al. 2013). By applying the logit transformation to Equation (4), we have


(4) $$\begin{array}{c} \pi_{j}^{i}=\mathrm{Pr}\left(y^{i}(j)|X^{i}(j);\theta_{i}\right)\\ =f\left(\theta_{i}^{⊤}X^{i}(j)\right)\\ =\frac{\;\text{exp}\;\left(\theta_{i}^{⊤}X^{i}(j)\right)}{1+\text{exp}\;\left(\theta_{i}^{⊤}X^{i}(j)\right)},\;i\in [1,N],\;j\in [1,T-1], \end{array}$$

(5) $$\begin{array}{c} \text{logit}(\pi_{j}^{i})=\text{log}(\frac{\pi_{j}^{i}}{1-\pi_{j}^{i}})\\ =\theta_{1}^{i}x_{1}(j)+\theta_{2}^{i}x_{2}(j)+\cdots +\theta_{N}^{i}x_{N}(j)+\theta_{0}^{i}. \end{array}$$

It can be seen that the logistic regression model (4) has a logit that is linear in $X^{i}(j)$.

#### 2.1.3 Estimation of the regression coefficients

The coefficient vector $\theta_{i}$ in Equation (4) is unknown. Here, we use the maximum likelihood approach and a regularized technique to fit the model (4) containing *N* variables (James et al. 2013). Since $y^{i}(j)$ follows the Bernoulli distribution, its probability function is given by $\mathrm{Pr}(y^{i}(j))={\left(\pi_{j}^{i}\right)}^{y^{i}(j)}{\left(1-\pi_{j}^{i}\right)}^{1-y^{i}(j)}$ for i∈[1,N], then we have the likelihood function as follows:


(6) $$L(\pi_{j}^{i})=\prod_{j=1}^{N}\mathrm{Pr}(y^{i}(j))=\prod_{j=1}^{N}{\left(\pi_{j}^{i}\right)}^{y^{i}(j)}{\left(1-\pi_{j}^{i}\right)}^{1-y^{i}(j)}.$$

The corresponding log-likelihood function is a function of the regression coefficient vector $\theta_{i}$ given by


(7) $$L(\theta_{i}|D_{i})=\sum_{j=1}^{N}\{y^{i}(j)\cdot \;\text{log}(\pi_{j}^{i})+[1-y^{i}(j)]\cdot \;\text{log}(1-\pi_{j}^{i})\}.$$

Clearly, by minimizing the negative of Equation (7), we can estimate the regression coefficient vector $\theta_{i}$. To avoid over-fitting, we add the elastic net penalty term (Zou and Hastie 2005) to Equation (7), and solve the regression coefficient vector $\theta_{i}$ according to the following regularized logistic regression model (Li and Liu 2020, 2022):


(8) $$\theta_{i}=\mathrm{arg min}\left\{-L(\theta_{i}|D_{i})+\lambda \left[\alpha {\left\|\theta_{i}\right\|}_{1}+(1-\alpha ){\left\|\theta_{i}\right\|}_{2}^{2}\right]\right\}.$$

Here ${\left\|\theta_{i}\right\|}_{1}=\sum_{k=1}^{N}|\theta_{k}^{i}|$ and ${\left\|\theta_{i}\right\|}_{2}^{2}=\sum_{k=1}^{N}\theta_{k}^{i}{}^{2}$ represent the *L*_1_-norm and the square of *L*_2_-norm, respectively. Here λ>0 is a tuning parameter used to balance the negative log-likelihood term and the elastic net penalty term, α∈[0,1] is used to shrink the estimated coefficient $\theta_{i}$ to control the sparsity of inferred network, i.e. the in-degree of the node *x_i_*.

#### 2.1.4 Aggregation of Boolean threshold function with regression coefficients

In this work, we adopt a synchronous update mode, i.e. all nodes evolve simultaneously at consecutive time points. Here, $\theta_{i}$ is obtained from Equation (8), the corresponding update scheme of *x_i_* in the form of Boolean threshold function can also be obtained. The details are as follows:


(9) $$\text{Update scheme}\;:=\{\begin{array}{cc} w_{k}=\theta_{k}^{i}\;\text{and}\;l_{k}=x_{k}, & \text{if}\;\theta_{k}^{i}>0,\\ w_{k}=0, & \text{if}\;\theta_{k}^{i}=0,\\ w_{k}=-\theta_{k}^{i}\;\text{and}\;l_{k}=\bar{x_{k}}, & \text{if}\;\theta_{k}^{i}<0. \end{array}$$

Then, the Boolean threshold function of *x_i_* is given as follows: where $\theta_{i}=-\theta_{0}^{i}-\sum_{k\in \{k|k\in [1,N],\theta_{k}^{i}<0\}}\theta_{k}^{i}$. Based on that, we have the following result.

Theorem 2.1. *The inequality* $w_{1}l_{1}+w_{2}l_{2}+\cdots +w_{N}l_{N}\geq \theta_{i}$*inEquation (10) is equivalent to* $\theta_{1}^{i}x_{1}(j)+\theta_{2}^{i}x_{2}(j)+\cdots +\theta_{N}^{i}x_{N}(j)+\theta_{0}^{i}\geq 0$*inEquation (5) under the given update scheme shown in Equation (9).*

(10) $$\begin{array}{c} x_{i}(t+1)=f_{i}(x_{1}(t),x_{2}(t),\ldots ,x_{N}(t))\\ =\{\begin{array}{cc} 1, & w_{1}l_{1}+w_{2}l_{2}+\cdots +w_{N}l_{N}\geq \theta_{i},\\ 0, & \text{otherwise}, \end{array} \end{array}$$

**Figure 1.**
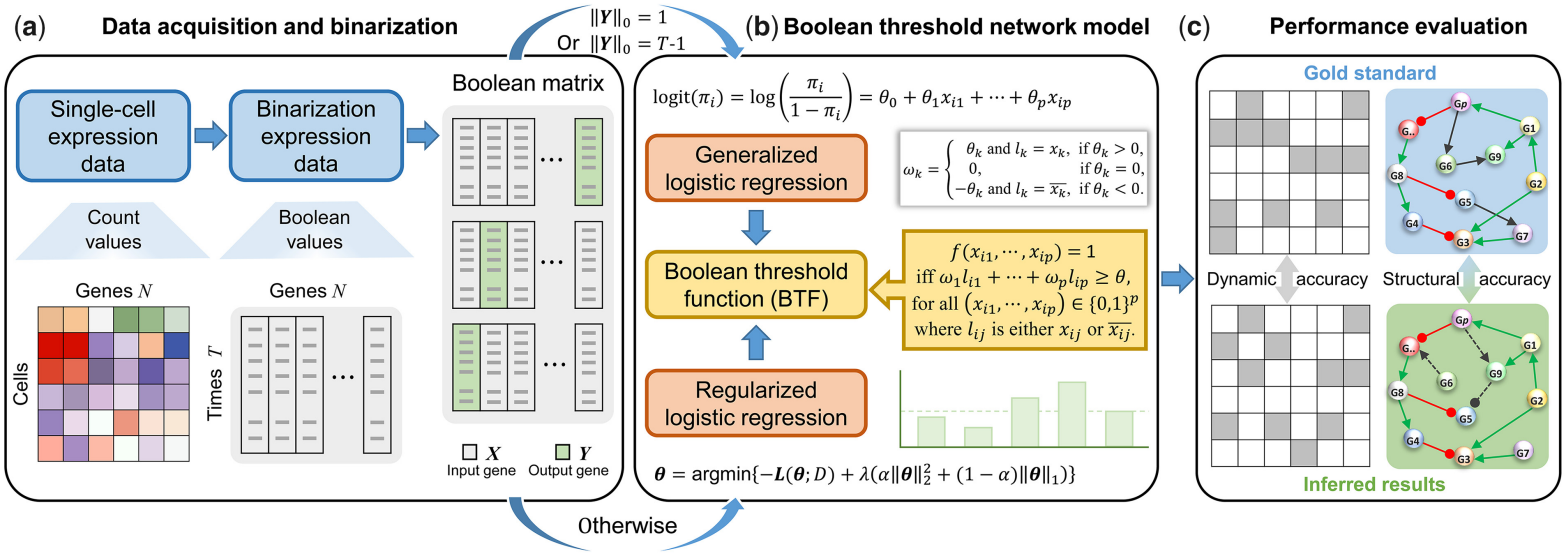
The framework of LogBTF for GRN inference from single-cell gene expression data, where ${\left\|Y\right\|}_{0}=\sum_{j=1}^{T-1}1(y_{j}\neq 0)$ is the *L*_0_-norm. (**a**) For single-cell gene expression data with corresponding pseudo-time series information, binarizing the count values into Boolean values (0 or 1). (**b**) Based on the binarized gene expression data, Boolean threshold network model is constructed by aggregating Boolean threshold function with logistic regression coefficients. (**c**) The inferred gene expression state and the reconstructed GRN are, respectively, compared with the original states and gold standard to evaluate the inference performance.

The proof of Theorem 2.1 can be available in Supplementary Equations (S1)–(S7). Therefore, we can use logistic regression model to estimate the parameters of Boolean threshold network model from the given dataset D. In this article, we call this aggregation strategy the LogBTF method and its novelty lies in the use of logistic regression and Boolean threshold function to construct a Boolean threshold network model. The framework of the LogBTF method for inferring GRN from single-cell gene expression data is shown in Fig. 1. Especially, the consistency between Boolean trajectory generated by the inferred network and the binarized time series gene expression data is characterized by the dynamical accuracy (DyAcc) metric, while the consistency between inferred GRN and the ground-truth GRN is characterized by the structural accuracy (StAcc) metric.

### 2.2 Datasets

First, an artificial Boolean value dataset with a ground-truth regulatory network is generated to evaluate the validity and accuracy of our proposed method. Then, considering that simulated single-cell gene expression data are the promising alternative approach for mimicking real data with their statistical properties and underlying biological relationships (Dibaeinia and Sinha 2020), so 15 simulated single-cell datasets from GeneNetWeaver (GNW) (Schaffter et al. 2011) and ten simulated single-cell datasets from SERGIO (Dibaeinia and Sinha 2020), guided by corresponding source networks (gold standard), are used in our experiments. Finally, three real scRNA-seq datasets mined from existing literature are also applied in our work. The details of all datasets are illustrated in Supplementary Table S1 and Supplementary Figs. S1–S3, in which SIGN is used to characterize the gold standard whether with signed edges (SIGN = 1, activation or inhibition) or not (SIGN = 0).

### 2.3 Data preprocessing

LogBTF method requires the state of each gene to be quantified as 0 (false/not expressed) or 1 (true/expressed). For the simulated or real single-cell expression data, no imputation is required, all dropouts are set to 0 and all non-zero counts are set to 1 regardless of the expression level (Qiu 2020). Moreover, for the bulk expression data additionally shown in the Supplementary Materials, the continuous gene expression values need to be pro-preprocessed by binarization. For each gene, its expression data at all time points can be seen as a one-dimensional vector, data binarization is the process of converting continuous data attribute values into finite interval sets, that is, 0 or 1 in Boolean modeling. The detailed implementation is presented in Supplementary Equation (S8).

### 2.4 Optimization of network topology

To overcome the multi-collinearity problem and detect reliable regulatory relationships, we propose an optimization strategy based on knowledge of the perturbation design matrix (Seçilmiş et al. 2022) as follows: first, a normal distribution matrix Σ with mean *μ *= 0 and variance $\sigma^{2}$ (enough small) is generated, and the dimension of Σ is the same as the dimension of binarized input matrix ***X***. Then, a new perturbation input matrix $\hat{X}≜X+\Sigma$ is obtained by adding the binarized input matrix and the random generated matrix. Based on the newly obtained input matrix $\hat{X}$, we apply the LogBTF method to estimate the regression coefficient ${\hat{\theta }}_{i}$ of the *i*-th target gene as follows:


(11) $${\hat{\theta }}_{i}={\left({\hat{\theta }}_{1}^{i},{\hat{\theta }}_{2}^{i},\ldots ,{\hat{\theta }}_{N}^{i},{\hat{\theta }}_{0}^{i}\right)}^{⊤},\;i\in [1,N].$$

Using the above strategy, the multi-collinearity problem is solved but the estimated value ${\hat{\theta }}_{i}$ is noisy, and if the resulting regression coefficients are used directly for GRN inference, some redundant edges are generated. To further optimize the inferred network topology, we first remove the influence of the added random tiny perturbation matrix Σ by setting the coefficient whose absolute value is less than the given *σ* value to 0. Then the remaining no-zero elements in the coefficient ${\hat{\theta }}_{i}$ are carried out to infer the potential GRN. Finally, we normalize the regulatory coefficient ${\hat{\theta }}_{i}$ of each gene by where ${\left\|{\hat{\theta }}_{i}\right\|}_{\infty }=\underset{1\leq k\leq N}{\max}|{\hat{\theta }}_{k}^{i}|$ is the $L_{\infty }$-norm. Thus, the contribution of all regulatory genes to the given target gene has a standard scale, with the strongest regulatory relationship being assigned 1, and the weakest being 0.


(12) $${\hat{\theta }}_{i}\leftarrow \frac{{\hat{\theta }}_{i}}{{\left\|{\hat{\theta }}_{i}\right\|}_{\infty }},$$

### 2.5 Parameter selection and performance evaluation

On one hand, the selection of optimal tuning parameters for our method is vital, the specific process can be found in Supplementary Equations (S9) and (S10). On the other hand, we define the DyAcc, StAcc, recall (Recal), precision (Pre), false positive rates (FPRs), *F*-measure, and the area under the ROC curve (AUC) (Bradley 1997) to evaluate the inferring performance of LogBTF and other eight inferring methods. At the same time, we assess the performance of LogBTF by evaluating the areas under the receiver operating characteristic (AUROC) (Hanley and McNeil 1982) and the precision–recall curve (AUPR). All corresponding definitions and calculation formulas of the above evaluation metrics and the differences between LogBTF and eight alternative GRN inference methods can be found in Supplementary Equations (S11)–(S13) and Supplementary Table S2.

## 3 Results

### 3.1 Simulated Boolean data

For ease of exposition, let $\left[w_{1}l_{1}+w_{2}l_{2}+\cdots +w_{N}l_{N}\geq \theta_{i}\right]$ denote the Boolean threshold function defined by


(13) $$\begin{array}{c} \left[w_{1}l_{1}+w_{2}l_{2}+\cdots +w_{N}l_{N}\geq \theta_{i}\right]\\ =\{\begin{array}{cc} 1, & w_{1}l_{1}+w_{2}l_{2}+\cdots +w_{N}l_{N}\geq \theta_{i},\\ 0, & \text{otherwise}. \end{array} \end{array}$$

According to Theorem 2.1, we find that $w_{1}l_{1}+w_{2}l_{2}+\cdots +w_{N}l_{N}\geq \theta_{i}$ in Equation (10) is equivalent to $\theta_{1}^{i}x_{1}(j)+\theta_{2}^{i}x_{2}(j)+\cdots +\theta_{N}^{i}x_{N}(j)+\theta_{0}^{i}\geq 0$ in Equation (5). We can therefore determine the coefficients $w_{1},w_{2},\ldots ,w_{N}$ and *θ_i_* in Equation (13) by estimating the regression coefficients. Specifically, $w_{k}=\theta_{k}^{i}$ if $\theta_{k}^{i}>0$; *w_k_*  =  0 if $\theta_{k}^{i}=0$; $w_{k}=-\theta_{k}^{i}$ if $\theta_{k}^{i}<0$ and $\theta_{i}=-\theta_{0}^{i}-\sum_{k\in \{k|k\in [1,N],\theta_{k}^{i}<0\}}\theta_{k}^{i}$.

First, we generated a set of synthetic expression data. Here, we first construct a BN with nine nodes as follows:


(14) $$\begin{array}{cc} x_{1}: & [x_{1}+x_{2}\geq 1],\\ x_{2}: & [\bar{x_{1}}+\bar{x_{4}}+\bar{x_{5}}+\bar{x_{9}}\geq 4],\\ x_{3}: & [\bar{x_{2}}+\bar{x_{5}}+\bar{x_{9}}\geq 3],\\ x_{4}: & [\bar{x_{2}}+x_{3}\geq 2],\\ x_{5}: & [\bar{x_{2}}+x_{3}+x_{5}\geq 3],\\ x_{6}: & [x_{9}\geq 1],\\ x_{7}: & [\bar{x_{5}}+2x_{6}+\bar{x_{9}}\geq 2],\\ x_{8}: & [x_{5}+x_{6}+5\bar{x_{7}}+4x_{8}+x_{9}\geq 5],\\ x_{9}: & [\bar{x_{6}}+\bar{x_{7}}\geq 2]. \end{array}$$

**Figure 2.**
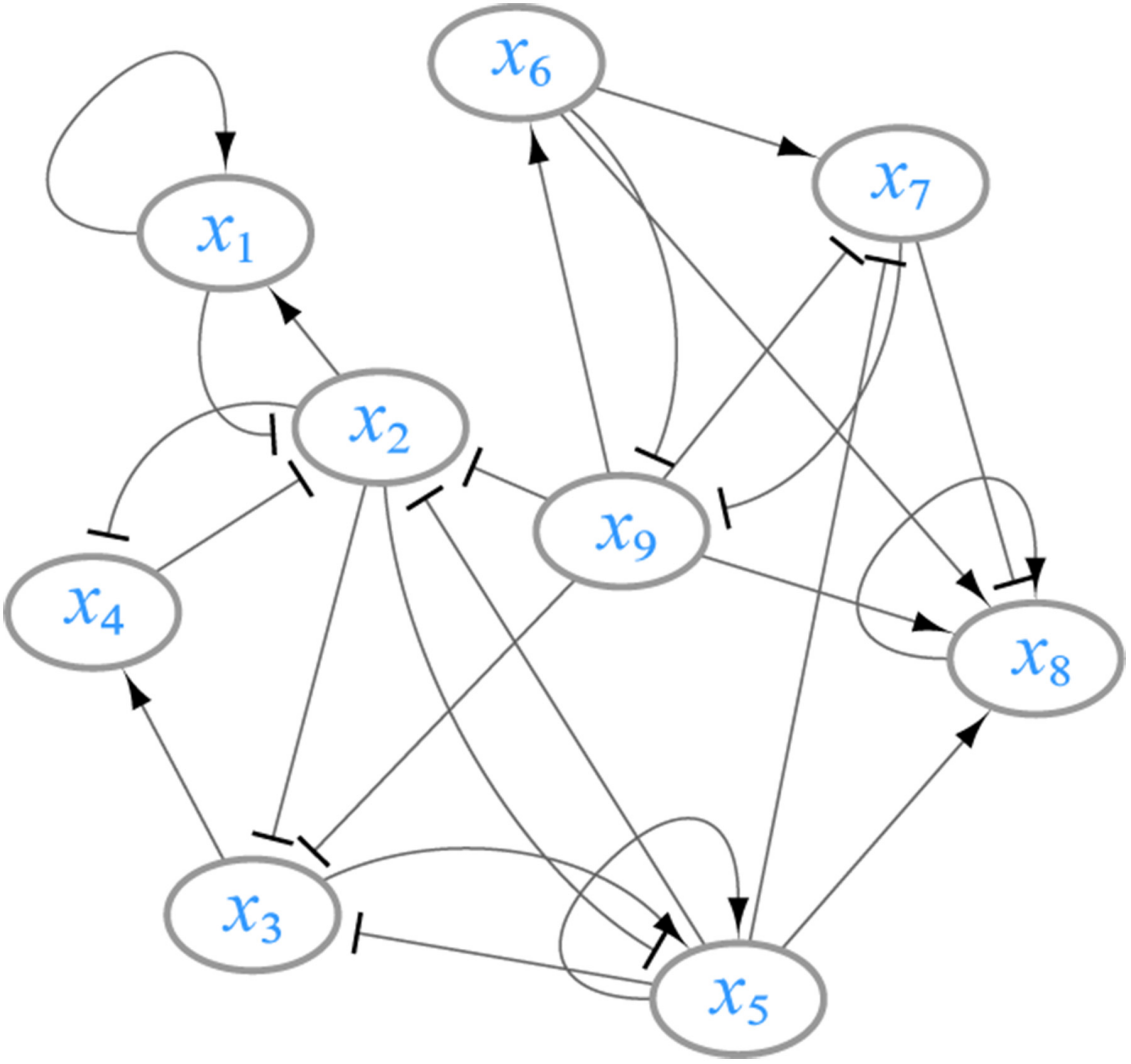
The synthetic Boolean threshold network, where each node *x_i_* has a state value of 1 (expressed) or 0 (not expressed). The directed links with “→” represent a positive regulation (activating) from *x_i_* to *x_j_*, and “⊣” represent a negative regulation (inhibiting).

Clearly, there are 2^9^ initial states. For each initial state, according to the Boolean threshold function in Equation (13), the state of each node at the next time point is obtained. Therefore, for each node *x_i_* with i∈[1,9], there are 512 observations in total. The Boolean threshold network corresponding to the BN in Equations (14) is shown in Fig. 2.

In the following, we use all possible initial states to form a state matrix at time *t*, and set this matrix as the input data ***X***. The state of the node at time *t *+* *1 is set as output ***y***. In this way, the input state matrix ***X*** is a full-rank matrix, which avoids the multi-collinearity problem between variables for coefficient estimation. Considering that the node size is only nine, it does not need to penalize the coefficients, so we employ the generalized linear regression (un-penalized logistic regression) model by setting *λ *= 0 in Equation (8). In this case, we obtain the inferred Boolean threshold network as shown in Supplementary Equation (S14). Concerning the threshold network structure in Fig. 2, the inferred BN in Equation (S14) and BN in Equation (14) are equivalent, which indicates that the LogBTF method is effective and efficient for inferring GRN from time series data.

In order to test the robustness of the LogBTF method, we investigate the tolerance of the model to data changes. Namely, we introduce noise into the simulated time series data by randomly flipping the state of each node with the probability of *δ* for δ∈{0%,1%,3%,5%}, respectively. As described in Table 1, all metrics remain stable when the noise increases from 1% to 5%, especially for Pre and FPR indexes. AUROC decreases from 0.920 (at δ=1%) to 0.880 (at δ=5%). The trend of AUPR under different noise levels is similar to that of AUROC. Remarkably, LogBTF can infer the correct topology of the idealized regulatory network under 0% noise. When the noise ratio increases to 5%, LogBTF can still infer the topology with StAcc = 0.926, Recal = 0.760, and *F*-measure = 0.864. In conclusion, it is robust and stable for LogBTF to adopt the Boolean threshold network model to improve the inference performance. Especially, we also explore the inference accuracy of the LogBTF method by randomly sampling a certain percentage of data from 512 observations, and the related results with discussions are given in Supplementary Fig. S4.

**Table 1.** The performance of LogBTF inferring the Boolean network with nine nodes under different noise levels.

Noise	AUROC	AUPR	StAcc	Recal	Pre	FPR	*F*-measure
δ=0%	1.000	1.000	1.000	1.000	1.000	0.000	1.000
δ=1%	0.920	0.943	0.951	0.840	1.000	0.000	0.913
δ=3%	0.900	0.928	0.938	0.800	1.000	0.000	0.889
δ=5%	0.880	0.913	0.926	0.760	1.000	0.000	0.864

### 3.2 Simulated single-cell data by GNW

#### 3.2.1 Case 1: SIGN = 1

**Figure 3.**
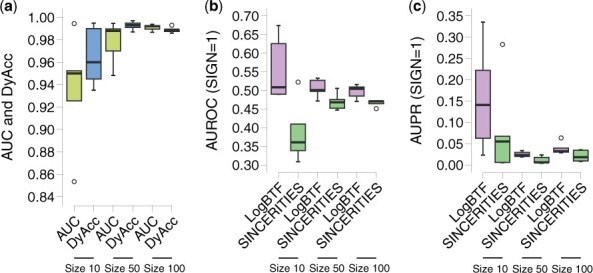
The prediction results of LogBTF method on simulated single-cell datasets in the case of SNGN = 1. (**a**) The AUC and DyAcc values of LogBTF method. (**b**) The AUROC and (**c**) AUPR comparison of LogBTF and SINCERITIES methods on three types of simulated datasets with different sizes.

In this experiment, we further simulated dropout datasets to investigate the GRN inference applicability of LogBTF to zero-inflated single-cell gene expression data (Chan et al. 2017). Namely, we induced dropout events at ∼20%−25% ratio for the data generated from GNW. For each sample, the expression values lower than the given threshold for each gene would be recorded as 0 according to a Binomial probability of 50% (Chen and Mar 2018). The expression value distributions and ground truth networks of all datasets are shown in Supplementary Figs. S1 and S2. We predict the expression state of each gene during all time points to investigate the performance of LogBTF in terms of predictive AUC value. Figure 3(a) shows that the means of the AUC value and DyAcc value on 15 simulated single-cell datasets are ˃0.92.

Considering that the SINCERITIES method is also functional in evaluating the inferred network from the aspect of activating and inhibitive regulations between regulator and target genes, we compare the AUROC and AUPR indexes of LogBTF with SINCERITIES under the situation of SIGN = 1. Figure 3(b) shows the results of the AUROC value comparing the LogBTF with the SINCERITIES method on three types of datasets of different sizes. We can see that our proposed LogBTF method performs better than SINCERITIES for all different network sizes. Also, there seems to be a trend that when the number of genes in the network increases, the difference between these two methods gradually decreases, under the premise that LogBTF is better than SINCERITIES. From Fig. 3(c), one can see that the AUPR values of LogBTF are significantly larger than SINCERITIES when the network size is small. While as the network size increases, our LogBTF method still shows strong superiority when compared with SINCERITIES, both from single experimental results and mean value. In addition, we also show the StAcc results in Supplementary Fig. S5, the larger the number of nodes in the network, the higher the StAcc value of our method.

#### 3.2.2 Case 2: SIGN = 0

**Figure 4.**
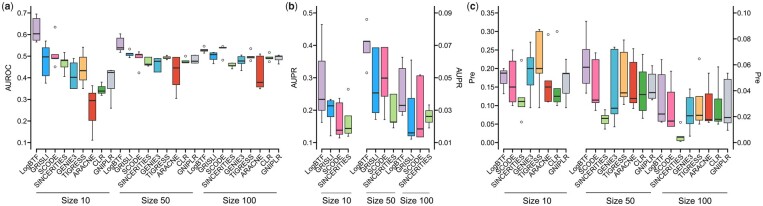
The comparing results of LogBTF with the other methods on three type datasets with different gene sizes from simulated single-cell data in the case of SNGN = 0. (**a**) The AUROC values. (**b**) The AUPR value. (**c**) The Pre value.

When omitting the activation or inhibition functions, we only study the regulatory relationships and the regulator/target roles among genes, i.e. SIGN = 0. In this case, the signature of regression or correlation coefficient does not work, which means that all coefficients can be taken as the measure like weights. Thus these nine GRN inference methods have a standard benchmark for comparisons. For more reliable performance validation, we further compare LogBTF with eight methods, including SINCERITIES that we just discussed, GRISLI, SCODE, GENIE3, TIGRESS, ARACNE, CLR, and GNIPLR. The comparing results of AUROC, AUPR, and Pre indexes on all available datasets are shown in Fig. 4, where each boxplot describes the statistical results from five independent runs with five simulated datasets under three different network size groups. The center line denotes the median, the lower and upper hinges, respectively, represent the 25th and 75th percentiles, the vertical lines express the 1.5 times interquartile range, and the dots outside the vertical lines are the outliers.

Figure 4(a) displays that our proposed LogBTF method is significantly superior to the other eight comparable methods in terms of AUROC evaluation with size 10 and size 50. In contrast, the performance of LogBTF is less satisfactory in the network with node size 100, where the SCODE achieves almost perfect inference. And, compared to all methods, ARACNE obtains the worst AUROC results on all simulated single-cell datasets. Although AUROC values are the classical choice for comparing methods, the AUPR value is more relevant for evaluating the network comparison (Chen and Mar 2018). Figure 4(b) shows the AUPR values among four inference methods specially developed for single-cell data, where the LogBTF method achieves the best results regardless of the network size of the dataset. In particular, when the size of network nodes is relatively large (such as size 50), our method has the largest mean AUPR and the smallest standard deviation.

The problem of GRN inference is a sparse prediction problem, which has a relatively low positive rate. In this case, Pre is a more valuable index because it measures the proportion of correctly inferred edges (Chen and Mar 2018). Figure 4(c) gives the Pre values comparing the inferred network relationship with sourced gold standards. Here we do not compare GRISLI method considering its source code does not output the Pre value metric. In the five datasets with size 10, the LogBTF method achieves lower (mean) Pre values than GENE3 and TIGRESS methods, but its standard deviation is smaller than theirs. As for the case of size 50 and size 100, compared with seven alternative methods, the mean Pre value of LogBTF is the highest. Especially, SINCERITIES obtains the worst Pre values among all methods on all 15 datasets, although whose standard deviation is the smallest.

All experimental results on datasets from GWN, including other estimation criteria (such as Recal, FPR, and *F*-measure), are available in Supplementary Tables S3–S6. They reflect the stability and applicability of the LogBTF method for different networks. Additionally, in order to study the applicability of our GRN inference method to bulk gene expression data, we also apply our LogBTF method to the synthetic bulk RNA-seq data from GNW (Schaffter et al. 2011). The results and discussion are available in Supplementary Figs. S6 and S7 and Supplementary Table S7. All results reflect the universality and applicability of the LogBTF method for large-scale networks.

### 3.3 Simulated single-cell data by SERGIO

To further verify the inference performance of LogBTF, ten single-cell gene expression datasets generated by SERGIO simulator (Dibaeinia and Sinha 2020) are employed in the numerical experiments as well. Especially, as a simulator for single-cell expression data guided by given GRNs, SERGIO can generate data that includes technical noise, outliers, and “dropout” and convert the data to Unique Molecular Identifier counts. In this part, we simulated the single-cell expression profiles of 20 and 100 genes with 5 kinds of cell numbers (10, 20, 30, 40, and 50) in each cell type. For the datasets with network size 20, we set the “dropout” parameters (i.e. percentile) ˃80, while setting the “dropout” parameters ˃50 for the datasets with network size 20. Thus, a total of ten simulation datasets are obtained, and the two prior networks (gold standard) are shown in Supplementary Fig. S3, respectively.

**Figure 5.**
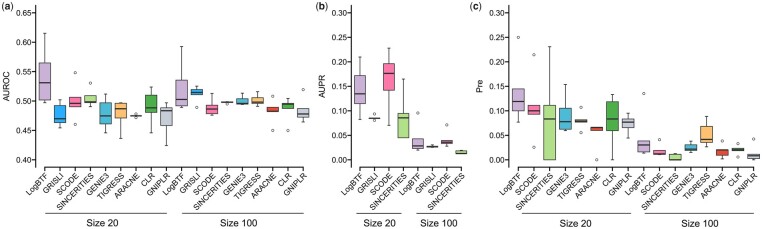
The comparison results of LogBTF with the other methods on two types of single-cell simulation datasets from SERGIO in the case of SNGN = 0. (**a**) The AUROC values. (**b**) The AUPR value. (**c**) The Pre value.

Next, we also conduct experiments using LogBTF method and eight other GRN inference methods on these ten simulated single-cell data. Previous experiments have demonstrated the inference performance of LogBTF on directed networks, so we will not repeat them here. Here we only comprehensively compare the network inference performances of different GRN inference methods in the case of SIGN = 0. Figure 5(a) shows that LogBTF achieves the best overall inference performance in terms of AUROC values regardless of network size 20 or 100. It also depicts that SINCERITIE ranks the second for the size 20 network and GRISLI ranks the second for the size 100 network. Moreover, consistent with the trend on the simulated dataset generated by GNW, the larger the network size is, the smaller the standard deviation of all methods will be.

In particular, compared with three popular methods (GRISLI, SCODE, and SINCERITIE) for inferring GRN from single-cell gene expression data, Fig. 5(b) gives the comparing results of AUPR values, which demonstrates that LogBTF outperforms GRISLI and SINCERITIE, but not better/competitive than SCODE on the datasets of size 20 network. As for the Precision (“Pre”) metric, just like the experiments in the last subsection, Fig. 5(c) illustrates that the proposed LogBTF method achieves higher inference accuracy than the other seven methods. In contrast, SINCERITIE performed poorly in terms of precision. As shown in Fig. 5, although the single-cell data generated by the SERGIO simulator has large dropouts, LogBTF is also effective in GRN inference and performs better than the other methods.

### 3.4 Real scRNA-seq data

**Figure 6.**
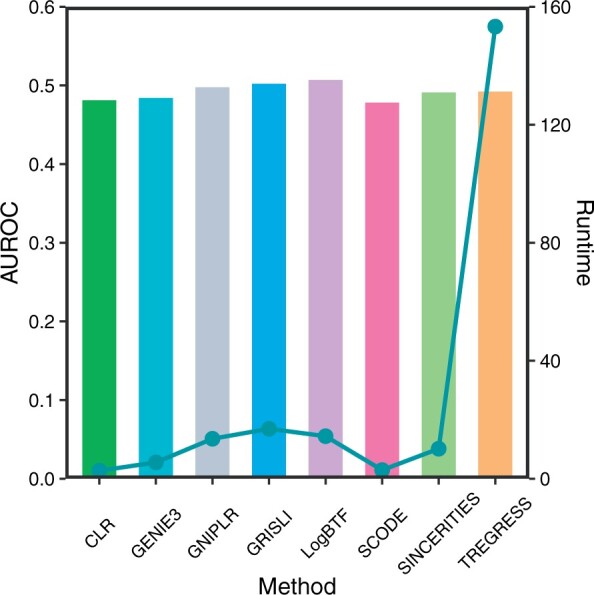
The performance comparisons among different GRN inference methods on Matsumoto dataset.

To evaluate the performance of the LogBTF method, we apply it to three retrieved real single-cell gene expression datasets. Considering that the known gold standard networks of Matsumoto and hHEP datasets do not underlie the activation or inhibition information, so we only investigate the performances in the case of SIGN = 0. Figure 6 shows the AUROC comparison result of LogBTF with the other seven methods on Matsumoto dataset, where LogBTF method behaves with higher AUROC values than those of the other seven methods and GRISLI shows a second-best AUROC. We remark that ARACNE performs extremely worst AUROC value (zero), here we do not show it in Fig. 6. So, ARACNE looks not appropriate in the case of scRNA-seq data with the pseudo-time, as mentioned in the prior work that it is not applied to the time course data (Cantone et al. 2009).

In addition, Fig. 6 also counts the runtimes, where the computation time of LogBTF is significantly smaller than GRISLI and TIGRESS. In theory, LogBTF and SINCERITIES have the same computational complexity, but we design a program to calculate more comprehensive indicators (including mean AUC and DyAcc) and consider the time evolution/update rules (in the form of Boolean threshold function equations) in each inference process, so the actual running time of LogBTF is almost double that of SINCERITIES. In particular, we note that TIGRESS is not suitable for large-scale network inference analysis due to the expensive computational time, though it can also obtain satisfactory inference results. Additionally, similar comparisons on hHEP dataset are given in Supplementary Fig. S8 and Supplementary Table S8. All the experiments are conducted on a workstation with two Xeon Gold 6226R CPUs and 256G of RAM.

Finally, we apply the LogBTF method to the LMPP scRNA-seq dataset with 31 genes and 531 pseudo-time points. As a result, LogBTF method infers 306 regulatory relationships with DyAcc = 0.676, taking 1.156 min. The inferred GRN shown in Supplementary Fig. S9 is represented by 31 genes with 166 activating and 140 inhibiting regulatory relationships. From the existing literature (Hamey et al. 2017), we found 72 regulatory relationships have been verified, with 40 activations and 21 inhibitions accurately inferred. Furthermore, we perform the network ontology analysis (Wang et al. 2011) to enrich the network biological significance of the regulatory relationship in the inferred GRN. The results can be found in Supplementary Table S9, which shows that the GRN inferred by the LogBTF method provides a reference for elaborating molecular regulatory mechanisms in biology.

## 4 Conclusions

In this study, we proposed the Boolean threshold network framework, named LogBTF, by aggregating regularized logistic regression with Boolean threshold function. LogBTF is a de novo GRN inference method, and it is also a logic model with explainability of the regulatory relationships among genes. First, we applied the logistic regression model to estimate the parameters of the Boolean threshold network model from given gene expression data and proved their equivalence in theory. Second, we designed an optimization strategy to handle the multi-collinearity problem caused by binarized data and applied a cross-validation procedure to select the optimal tuning parameters. Finally, we conducted extensive experiments with the single-cell gene expression datasets of simulated, *in silico* and real and compared the performance of our method with those of eight well-known existing inference methods. In particular, our method significantly outperformed them in terms of AUROC, AUPR, Precision, and other evaluation criteria. Apparently, these results indicate that the proposed approach is a promising tool for accurate regulatory networks from time series single-cell gene expression data. Although the LogBTF method increases computation cost by utilizing cross-validation to choose the optimal parameters, it is possible to reduce the running time by parallel implementation, which will be included in our future study. Another future direction is to infer GRNs by constructing an asynchronously updated Boolean threshold network model and then comparing its results on the network dynamics with that of the synchronous update model.

## Supplementary Material

btad256_Supplementary_Data
