# Supplementary material for: LogBTF: gene regulatory network inference using Boolean threshold network model from single-cell gene expression data
Source: Bioinformatics. 2023 Apr 20;39(5):btad256. doi: 10.1093/bioinformatics/btad256 (PMC10172039; doi:10.1093/bioinformatics/btad256)
Supplement: btad256_Supplementary_Data [file btad256_supplementary_data.pdf]

Supplementary Materials for LogBTF: Gene regulatory network inference using Boolean threshold network model from single-cell gene expression data

Lingyu Li[a,b], Liangjie Sun[b], Guangyi Chen[a], Chi-Wing Wong[b], Wai-Ki Ching[b,*], Zhi-Ping Liu[a,**]

[a]*Department of Biomedical Engineering, School of Control Science and Engineering, Shandong University, Jinan, Shandong 250061, China*
[b]*Advanced Modeling and Applied Computing Laboratory, Department of Mathematics, The University of Hong Kong, Pokfulam Road, Hong Kong SAR, China*

1. The proof of Theorem 2.1

Theorem 2. 1. *The inequality* $w_1 l_1 + w_2 l_2 + \cdots + w_N l_N \geq \theta_i$ *in*

$$x_i(t+1) = f_i(x_1(t), x_2(t), \ldots, x_N(t)) = \begin{cases} 1, & w_1 l_1 + w_2 l_2 + \cdots + w_N l_N \geq \theta_i, \\ 0, & \textit{otherwise}, \end{cases} \tag{S1}$$

is equivalent to $\theta^i_1 x_1(j) + \theta^i_2 x_2(j) + \cdots + \theta^i_N x_N(j) + \theta^i_0 \geq 0$ *in*

$$logit(\pi^i_j) = \log\left(\frac{\pi^i_j}{1-\pi^i_j}\right) = \theta^i_1 x_1(j) + \theta^i_2 x_2(j) + \cdots + \theta^i_N x_N(j) + \theta^i_0. \tag{S2}$$

under the given update scheme

$$\textit{Update scheme} := \begin{cases} w_k = \theta^i_k \textit{ and } l_k = x_k, & \textit{if } \theta^i_k > 0, \\ w_k = 0, & \textit{if } \theta^i_k = 0, \\ w_k = -\theta^i_k \textit{ and } l_k = \overline{x_k}, & \textit{if } \theta^i_k < 0. \end{cases} \tag{S3}$$

Proof. For a fixed node x_i, if its status at the next time point will be evolved to 1, i.e., the Boolean threshold function takes the "True" state, then it holds

$$w_1 l_1 + w_2 l_2 + \cdots + w_N l_N \geq \theta_i. \tag{S4}$$

According to Equation (S3), Equation (S4) can be written as

$$\sum_{j\in\{k|k\in[1,N],\theta^i_k>0\}} \theta^i_k x_k - \sum_{k\in\{k|k\in[1,N],\theta^i_l<0\}} \theta^i_k \overline{x_k} \geq -\theta^i_0 - \sum_{k\in\{k|k\in[1,N],\theta^i_k<0\}} \theta^i_k, \tag{S5}$$

Since $1 - x_k = \overline{x_k}$, so we have

$$\sum_{k\in\{k|k\in[1,N],\theta^i_k>0\}} \theta^i_k x_k - \sum_{k\in\{k|k\in[1,N],\theta^i_k<0\}} \theta^i_k (1-x_k) \geq -\theta^i_0 - \sum_{k\in\{k|k\in[1,N],\theta^i_k<0\}} \theta^i_k. \tag{S6}$$

Clearly, by a simple operation, Equation (S6) becomes

$$\theta^i_1 x_1(j) + \theta^i_2 x_2(j) + \cdots + \theta^i_N x_N(j) + \theta^i_0 \geq 0. \tag{S7}$$

The proof is thus completed. □

* Corresponding author
** Corresponding author
Email addresses: lingyuli@mail.sdu.edu.cn (Lingyu Li), u3006446@connect.hku.hk (Liangjie Sun), guangyi_chen.jn@foxmail.com (Guangyi Chen), cwwongab@hku.hk (Chi-Wing Wong), wching@hku.hk (Wai-Ki Ching), zpliu@sdu.edu.cn (Zhi-Ping Liu)

2. The details of all datasets and preprocessing implementation

First, one artificial Boolean value dataset with a standard gene regulatory network is generated to evaluate the applicability, robustness and accuracy of our proposed LogBTF method.

Then, fifteen simulated datasets were generated using GeneNetWeaver (GNW) (Schaffter et al., 2011), a well-known simulation tool to generate gene expression data and "gold standard" networks for evaluating different GRN inference methods (Chen & Mar, 2018).

1. On one hand, noise-free "bulk" gene expression data with the corresponding source network (gold standard) from real *E.coli* and *Yeast* species are generated. Namely, there are three types of available datasets according to the number of genes (size 10, size 50 and size 100). Each type contains five *insilico* networks (*Ecoli*1, *Ecoli*2, *Yeast*1, *Yeast*2 and *Yeast*3) with 21 time points with the same step.
2. On the other hand, the aim of this study is to test the applicability of network inference methods to single-cell data, while drop-out events are one of the most important features of single-cell data. Therefore, we artificially induced the dropout data with excessive zero counts to the data generated from GNW to mimic the characteristics of single-cell experimental data (Chen & Mar, 2018), which is widely used to benchmark numerous GRN inference methods (Dibaeinia & Sinha, 2020).
3. Specifically, for each gene, we cluster its expression across cell samples into two clusters using K-means algorithm. For each sample, if the gene's expression was closer to the smaller cluster center, it would be replaced according to a Binomial probability of 0.5 (i.e., inducing drop-out where the resulting value was now either 0 or the original data point).
4. Additionally, the distributions of gene expression value for both simulated single-cell data and simulated bulk gene expression data used in this work are shown in Figure S1, and their corresponding networks are shown in Figure S2.

Next, in our study, we also apply the single-cell simulator called SERGIO (Dibaeinia & Sinha, 2020) to simulate the single-cell expression data based on the given GRN information.

1. SERGIO simulates each cell differential trajectory state according to stochastic differential equations (SDE), and takes the simulation results at the steady-state time as the gene expression profiles. Then, the cell splicing step is simulated and multiple transformations are performed to approximate the real state of the cell.
2. In particular, the simulator generates the data that includes technical noise, outliers and 'dropout' and converts it to Unique Molecular Identifier (UMI) counts. In this way, the simulated data to an extent matches published real scRNA-seq datasets (Dibaeinia & Sinha, 2020).
3. Here, we simulate the expression profiles of 20 and 100 genes on one cell type with five kinds of cell numbers (10, 20, 30, 40 and 50). For the datasets with network size 20, we set the 'dropout' parameters as $shape$ = 6.5, $percentile$ = 83, while $shape$ = 6.5, $percentile$ = 52 for the datasets with network size 20.
4. Thus, a total of ten simulated datasets are obtained, and the two provided networks (gold standard) are respectively shown in Figure S3, where each type of cell is in a time series state.

Finally, we collected three retrieved real single-cell gene expression datasets: Matsumoto (Treutlein et al., 2016), hHEP (Camp et al., 2017) and LMPP (Hamey et al., 2017).

1. The first raw dataset is real Matsumoto scRNA-Seq data obtained by examining direct reprogramming from mouse embryonic fibroblast (MEF) into myocytes at days 0, 2, 5 and 22. This dataset contains 373 cells with corresponding pseudo-temporal information and expression dynamics.
2. The second real specific human mature hepatocytes (hHEP) data, derived from a scRNA-seq experiment that induces pluripotent stem cells (iPSCs) to differentiate into hepatocyte-like cells in two-dimensional culture, is used as the fourth dataset in this paper. In this dataset, it contains 425 scRNA-seq measurements from multiple time points: days 0, 6, 8, 14 and 21, and the pseudo-time was obtained using Slingshot with cells measured on day 0 as the starting cluster and the cells measured on 21 (mature hepatocytes) as the ending cluster.
3. The last dataset we used is the data of transforming single hematopoietic stem cells (HSCs) into lymphoid-primed multipotent progenitors (LMPP). In this dataset, cells belonging to this branch were ordered into the pseudo-time differentiation trajectories to allow investigation of gene expression dynamics during HSCs differentiation.

4. By the way, these three datasets with their ground truth networks can be acquired from the listed references.

The details of all datasets used in our experiments are illustrated in Table S1, in which we use SIGN to characterize the gold standard whether with signed edges (SIGN = 1, activation or inhibition) or not (SIGN = 0).

Table S1 The details of all datasets used in our experiments, where '–' indicates that the value is set artificially.

Experiments	Datasets	# time point	# of nodes	Signed edges	References
Boolean dataset and network	Artificial data	–	9	SIGN = 1	–
Simulated single-cell dataset and *insilico* networks by GNW	Ecoli1 data	21	Size10, Size50, Size100	SIGN = 1	(Schaffter et al., 2011)
	Ecoli2 data	21	Size10, Size50, Size100	SIGN = 1	
	Ecoli3 data	21	Size10, Size50, Size100	SIGN = 1	
	Yeast1 data	21	Size10, Size50, Size100	SIGN = 1	
	Yeast2 data	21	Size10, Size50, Size100	SIGN = 1	
Simulated single-cell dataset and underlying GRN by SERGIO	Dataset1	10, 20, 30, 40, 50	Size20	SIGN = 1	(Dibaeinia & Sinha, 2020)
	Dataset2	10, 20, 30, 40, 50	Size100	SIGN = 1	
Real scRNA-seq dataset with pseudo-time and ground truth metwork	Matsumoto data	373	100	SIGN = 0	(Treutlein et al., 2016)
	hHEP data	425	948	SIGN = 0	(Camp et al., 2017)
	LMPP data	531	31	SIGN = 1	(Hamey et al., 2017)

3. Data discretization using K-means algorithm

For the simulated or real-world single-cell expression data, no imputation is required, all dropouts are set to 0 and all non-zero counts are set to 1s regardless of the expression level (Qiu, 2020). For the simulated or real-world bulk expression data, each gene's expression data at all time points can be seen as a one-dimensional vector. We apply K-means algorithm to split the given data into two clusters with $K = 2$ in an unsupervised way (Müssel et al., 2016).

1. First, it randomly picks two initial cluster centers from the original data and then assigns the remaining points to the closest cluster center.
2. Then, it updates this cluster progress by calculating the mean of the data in each cluster during several consecutive cycles, redefines the cluster centers to these mean values and reassigns all data points to the closest updated cluster center by minimizing the sum over the squared distance of the data points to their respective cluster center, i.e.,
$$\sum_{j=1}^{2} \sum_{x_i \in \text{Cluster}_j} (x_i - x_j^*)^2, \tag{S8}$$
where Cluster_j is the j-th cluster, x_i is one value of the input one-dimensional gene expression vector, x_j^* is the center of the j-th cluster.
3. Finally, the values belonging to the cluster with the smaller center are set to 0, and the values belonging to the larger center are set to 1.

4. The necessity of network topology optimization

The binarized Boolean value data obtained by the K-means algorithm may suffer from the multi-collinearity problem if the number of time points is small. For example, the binarized data of two different genes are same or differ by a minus sign. In this case, using logistic regression to estimate the parameters of the Boolean threshold network model, it may happen that only one gene is selected from a group of genes with high correlation (positive or negative correlation), so that the coefficient of the selected gene is a real number, but the coefficients of other genes are labeled "NA". Obviously, in this case, we cannot determine whether the selected gene is a true regulator. To overcome the multi-collinearity problem and detect reliable regulatory relationships, we need to propose an optimization strategy. In our paper, it is based on knowledge of the perturbation design matrix (Seçilmiş et al., 2022).

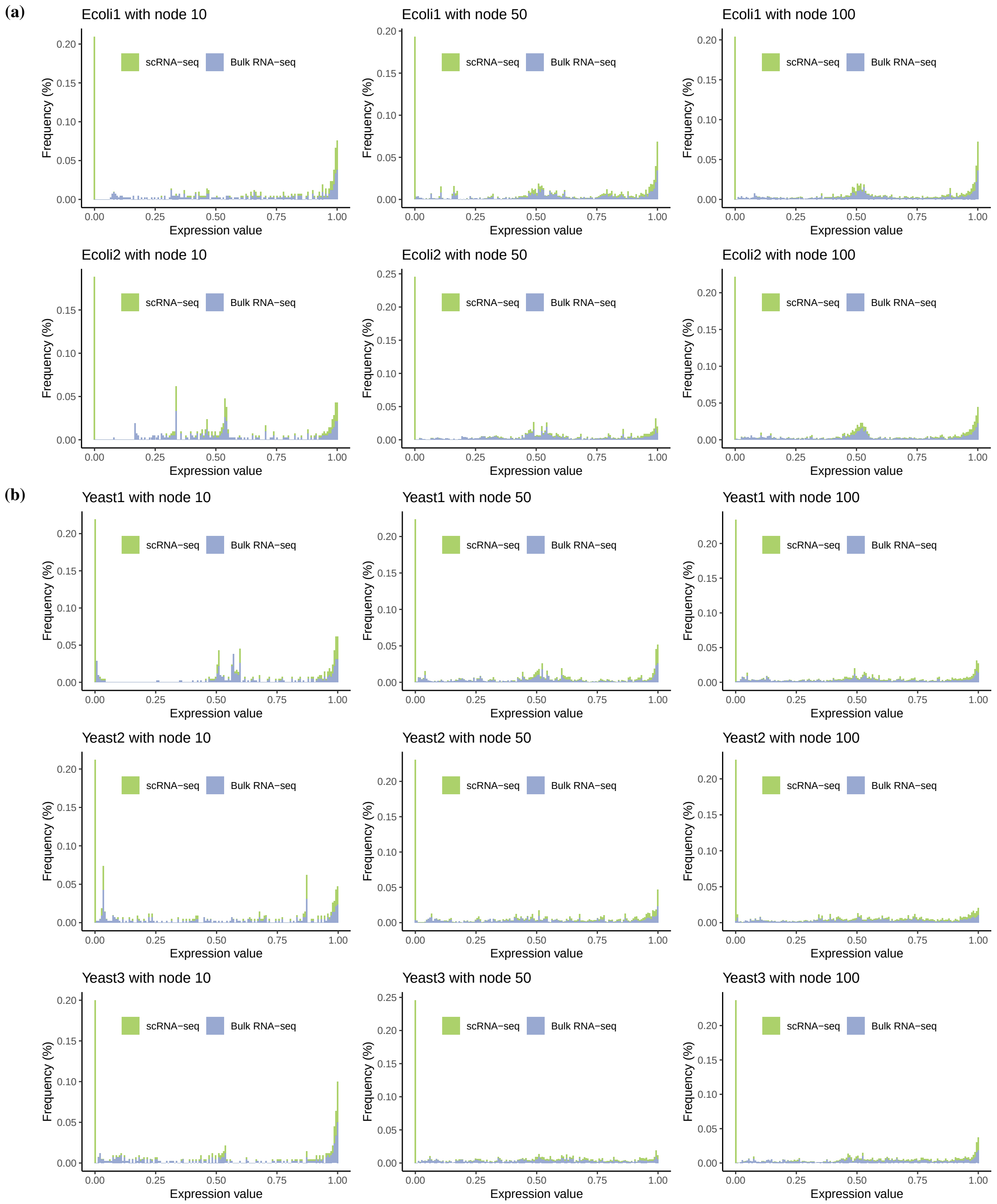

Figure S1 The distributions of gene expression value for both simulated single-cell and simulated "bulk" data. (a) Six *insilico* datasets for Ecoli and (b) Nine *insilico* datasets for Yeast, generated from two 10-gene, two 50-gene and two 100-gene networks.

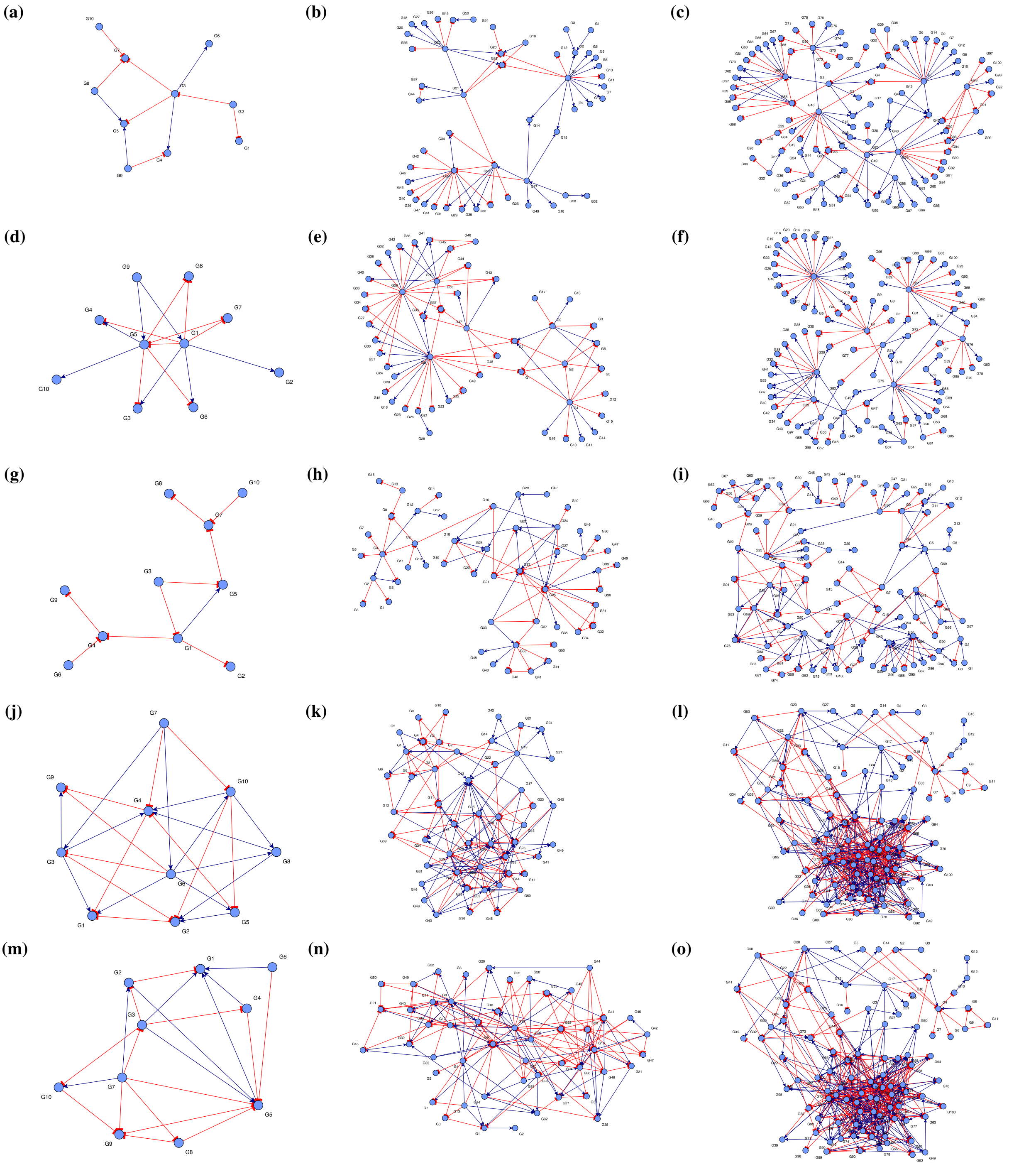

Figure S2 The ground truth networks of fifteen simulated (single-cell and "bulk") gene expression datasets. Three networks of in silico datasets from 10-gene, 50-gene and 100-gene for Ecoli 1 (a)-(c); Ecoli 2 (d)-(f); Yeast 1 (g)-(i); Yeast 2 (j)-(l) and Yeast 3 (m)-(o).

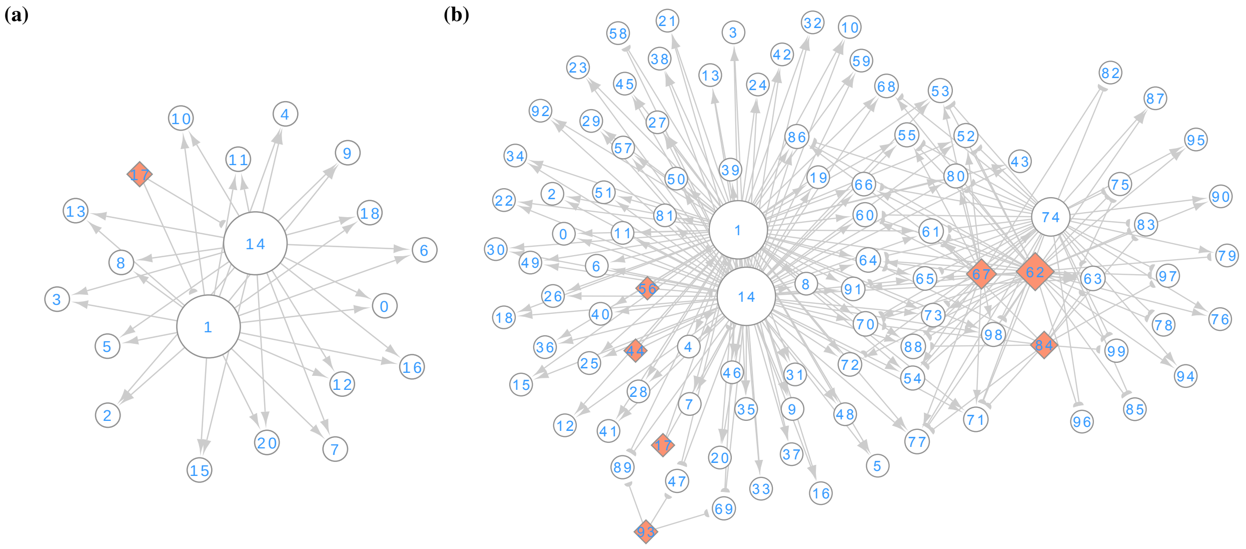

Figure S3 The structure of two gene regulatory networks used in SERGIO simulator (Dibaeinia & Sinha, 2020). These figures were generated using GNW package (Schaffter et al., 2011). As mentioned, all the auto-regulatory edges as well as cycles were removed prior to feeding networks to SERGIO although they are present in these figures. (a) The network 1, sampled from E.coli, contains 20 genes and 36 regulatory edges. (b) The network 2, sampled from E.coli, contains 100 genes and 258 regulatory edges.

5. Selection of tuning parameters

The selection of optimal tuning parameters for our method is vital in performing inferring GRNs. Without loss of generality, for $\boldsymbol{\theta}_i = \arg\min \left\{ -\mathscr{L}(\boldsymbol{\theta}_i|\mathscr{D}_i) + \lambda \left[\alpha \|\boldsymbol{\theta}_i\|_1 + (1-\alpha)\|\boldsymbol{\theta}_i\|_2^2 \right] \right\}$, we set α to a fixed value of 0.5 (Li & Liu, 2020), and then implement the K-fold cross-validation (CV) procedure in combination with search algorithms to obtain the optimal tuning parameter λ (James et al., 2013).

1. At first, the whole dataset is randomly split into K folds with approximately equal sample sizes, and each fold contain the corresponding responding input genes at t time point and output genes at $t+1$ time point.
2. Then, the $K-1$ folds data constitutes the training dataset used to train and fit the Boolean threshold network model, and the remaining fold as the test dataset used to evaluate the inference performances of the trained model by calculating the mean squared error (MSE):

$$\text{MSE} = \frac{1}{T-1} \sum_{j=1}^{T-1} [y^i(j) - f_i(x_1(j), x_2(j), \cdots, x_N(j))]^2, \quad i \in [1, N], \tag{S9}$$

   for each value of λ over the a range of different λ values. The procedure is performed K times and finally results in K estimates of the test error.
3. The final K-fold CV estimate value is defined as the average value of all MSE errors, that is,

$$\text{CV}(\lambda) = \frac{1}{K} \sum_{i=1}^{K} \text{MSE}_i. \tag{S10}$$

4. Finally, the optimal choice of regularization parameter λ can be obtained by maximizing Equation (S10).

Note that the choice of K depends on the size of the training dataset (Liang et al., 2013). In our experiments, if the network size is relatively small, we set $K = N$ and perform leave-one-out cross-validation (LOOCV) to fit the Boolean threshold network model N times. LOOCV uses almost all data, which guarantees the bias of the model to be smaller. But if the network size is extremely large, the computational cost of LOOCV can be expensive. If the network size is more than 500, we set $K = 10$ to conduct typical 10-fold CV to save the running times (James et al., 2013).

6. Evaluation criteria of inference

6.1. Dynamical accuracy metrics

Most of the existing algorithms for reconstructing BNs cannot accurately give Boolean function during each update status (Liu et al., 2021; Barman & Kwon, 2020; Schwab et al., 2021; Barman & Kwon, 2018). However, the LogBTF method can represent the corresponding Boolean threshold function for each gene. The logistic regression coefficient-based Boolean threshold function can be applied to evaluate the dynamical accuracy (DyAcc) by comparing the Boolean trajectory generated by the inferred network and the binarized time-series gene expression data.

Let $\tilde{x}_i(t)$ be the predicted Boolean value of gene x_i at time t in the inferred Boolean threshold network, we define the DyAcc of an inferred network using the average gene-wise consistency/similarity between the Boolean trajectories of the observed gene expression $x_i(t)$ and the estimated gene expression $\tilde{x}_i(t)$ over all N genes as follows:

$$\text{DyAcc} = \frac{1}{N(T-1)} \sum_{i=1}^{N} \sum_{t=2}^{T} \mathcal{I}(x_i(t) = \tilde{x}_i(t)), \tag{S11}$$

where $\mathcal{I}(\cdot)$ is the indicator function as follows:

$$\mathcal{I}(\cdot) = \begin{cases} 1, & x_i(t) = \tilde{x}_i(t), \\ 0, & \text{otherwise}. \end{cases} \tag{S12}$$

6.2. Structural accuracy metrics

Mathematically speaking, the gold standard or ground-truth GRN can be represented by a directed graph G with a gene set V and an edge set E, i.e., $G = (V, E)$. Correspondingly, the inferred GRN by Boolean threshold network model can also be characterized using $\tilde{G} = (\tilde{V}, \tilde{E})$. An important evaluation metric for the inference performance of a method is measuring the consistency/similarity between graph G and graph $\tilde{G}$. As usual, we compute the numbers of true positive (TP) edges, true negative (TN) edges, false positive (FP) edges and false negative (FN) edges by comparing the regulatory edges on a graph G and graph $\tilde{G}$ respectively. Structural accuracy (StAcc) is the ratio of correct predictions out of all predictions as follows:

$$\text{StAcc} = \frac{TP + TN}{TP + TN + FP + FN}. \tag{S13}$$

Especially, when considering GRN with signed edges, a true positive prediction refers to the correct prediction of an edge and its sign.

6.3. Other evaluation criteria

Moreover, we also use the following measures: recall (Recal, $TP/(TP + FN)$), precision (Pre, $TP/(TP + FP)$), false positive rates (FPR, $FP/(FP + TN)$), F-measure ($2TP/(2TP + FP + FN)$) and the area under the ROC curve (AUC) (Bradley, 1997) to evaluate the inferring performance of LogBTF and other inferring methods, including SINCERITIE (Papili Gao et al., 2018) and GNIPLR (Zhang et al., 2021) (regression-based method), GRISLI (Aubin-Frankowski & Vert, 2020) and SCODE (Matsumoto et al., 2017) (ordinary differential equation methods), GENIE3 (Huynh-Thu et al., 2010) and TIGRESS (Haury et al., 2012) (miscellaneous methods), ARACNE (Margolin et al., 2006) and CLR (Faith et al., 2007) (correlation-based methods). At the same time, we assess the performance of LogBTF by evaluating the areas under the receiver operating characteristic (AUROC) (Hanley & McNeil, 1982) and the precision-recall curve (AUPR). For the GRISLI method, we apply the source MATLAB code, which directly outputs the AUROC and AUPR values.

It is worth noting that SCODE, GENIE3, TIGRESS, ARACNE, CLR and GNIPLR methods cannot give a certain number of inferred edges. In order to guarantee a fair comparison, we select the same number of regulatory edges with the top κ edges from the ranked list output of SCODE, GENIE3, TIGRESS, ARACNE, CLR and GNIPLR methods, where κ is the number of links inferred from the LogBTF method. On the other hand, the SINCERITIE method can infer the number of edges. In summary, the differences between LogBTF and seven alternative gene regulatory network inference methods are shown in Table S2.

Table S2 The comparison with other network inferring models.

Method	Dlink	SIGN	InferBas	DyAcc	StAcc	Update rule	Gene status	# of in-degree	# of RegShips	Computational complexity
CLR	✘	✘	CorCoef	✘	T-based	✘	✘	✘	✘	$\mathcal{O}(N^2)$
ARACNE	✘	✘	CorCoef	✘	T-based	✘	✘	✘	✘	$\mathcal{O}(N^3)$
TIGRESS	✔	✘	Weights	✘	T-based	✘	✘	✘	✘	$\mathcal{O}(RN^{3/2}T), R = 1000$
GENIE3	✔	✘	Weights	✘	T-based	✘	✘	✘	✘	$\mathcal{O}(RN^{3/2}T \log T), R = 1000$
GNIPLR	✔	✘	F-test	✘	T-based	✘	✘	✘	✘	$\mathcal{O}(N^2T)$
SCODE	✔	✘	CoefMat	✘	T-based	✘	✘	✘	✘	$\mathcal{O}(RNT + N^2)$
GRISLI	✔	✘	CoefMat	✘	–	✘	✘	✘	✘	–
SINCERITIES	✔	✔	RegCoef	✘	✔	✘	✘	✔	✔	–
LogBTF	✔	✔	RegCoef	✔	✔	✔	✔	✔	✔	–

*Abbreviation: Dlink – Directed links; InferBas – Inference basis; CorCoef – Correlation coefficients; CoefMat – Coefficient Mtarix; RegCoef – Regression coefficients; T-based – Threshold-based; RegShips – regulatory relationships. *The Computational complexity of gene regulatory network inference methods wit respect to: N – the number of genes; R – the number of iterations; T – the number of time points.

7. Results on artificial Boolean data

7.1. Ground truth and inferred Boolean threshold network

By employing the generalized linear regression (un-penalized logistic regression) model, we obtained the inferred Boolean threshold network as follows:

$$\begin{aligned}
x_1 &: \quad [56.7x_1 + 56.7x_2 \geq 28.4],\\
x_2 &: \quad [54.7\overline{x_1} + 54.7\overline{x_4} + 54.7\overline{x_5} + 54.7\overline{x_9} \geq 191.8],\\
x_3 &: \quad [56.6\overline{x_2} + 56.6\overline{x_5} + 56.6\overline{x_9} \geq 141.7],\\
x_4 &: \quad [56.7\overline{x_2} + 56.7x_3 \geq 85.1],\\
x_5 &: \quad [56.6\overline{x_2} + 56.6x_3 + 56.6x_5 \geq 141.7],\\
x_6 &: \quad [57.1x_9 \geq 28.6],\\
x_7 &: \quad [56.6\overline{x_5} + 113.7x_6 + 56.6\overline{x_9} \geq 85.1],\\
x_8 &: \quad [53.6x_5 + 53.6x_6 + 216.2\overline{x_7} + 162x_8 + 53.6x_9 \geq 188.5],\\
x_9 &: \quad [56.7\overline{x_6} + 56.7\overline{x_7} \geq 85].
\end{aligned} \tag{S14}$$

Here all coefficients are kept to one decimal place in this experiment for the convenience of representation.

7.2. Inference accuracy results under random sampling

In order to test the high accuracy or effectiveness of the LogBTF method, we randomly sample a certain percentage (abbreviated as "percent") of data from the 512 observations, in which each group of data is randomly sampled ten times. We calculated the average AUC value of the gene expression status obtained by the Boolean threshold network model. At the same time, we calculated the minimum sample size required to infer the state of each gene at different time points when AUC=1, and the results are shown in Figure S4.

Here we only give the sample size required to infer two consecutive AUC values of 1 for each node. We consider this percentage to be the minimum number of samples required to correctly infer the state of the node. From Figure S4, we see that it only needs 2% data to infer the expression state of x_6 that evolves completely. For gene x_8, although it may need approximate 30% data to reach its inferred AUC value equal to 1, we can see that its AUC value is already very close to 1 when the percentage is around 10%. The other seven genes also show that our method does not require data at all time points to make accurate predictions about the dynamic expression behavior of genes.

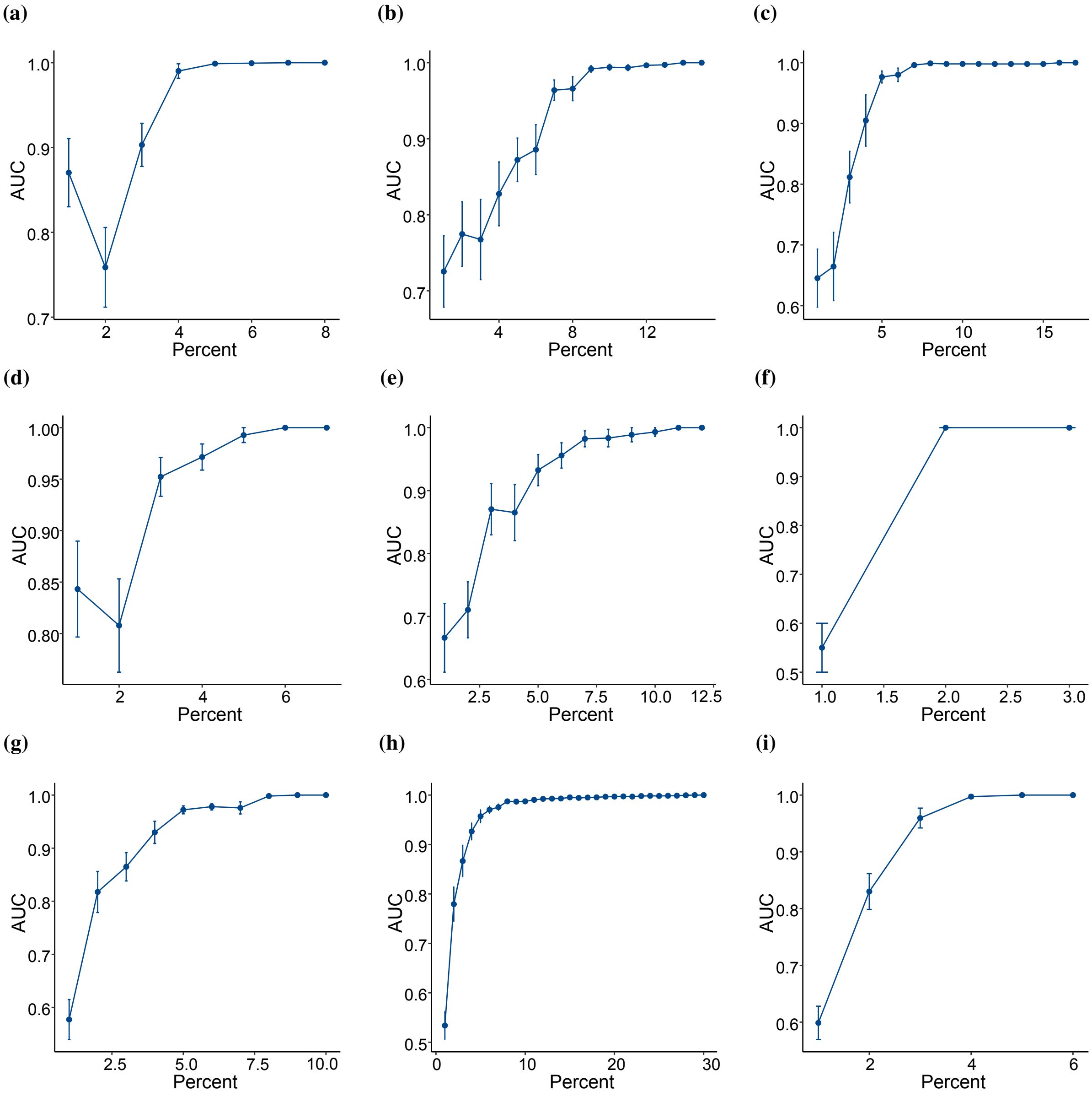

Figure S4 The AUC value of ten times random sampling under different percentages, where the polyline drawing ends if the AUC value of each node did not change with the increase of the sampling percentage. (a) x_1. (b)x_2. (c) x_3. (d) x_4. (e) x_5. (f) x_6. (g) x_7. (h) x_8. (i) x_9.

8. Results on simulated single-cell data by GWN

In this experiment, we use the leave-one-out cross-validation (LOOCV) method (James et al., 2013) to select the optimal tuning parameter and investigate the performance of LogBTF method. Figure S5 shows the experiment results. We can see that the StAcc values of LogBTF are larger than 0.77 when the network sizes are 50 and 100. Clearly, LogBTF method shows strong superiority when the network size is 100, compared with size 10 and size 50, both from single experimental results and mean value. Furthermore, our LogBTF method performs well both for directed GRN inference (SIGN = 1) and undirected internship inference (SIGN=0), and the larger the number of nodes in the network, the higher the StAcc value of our method.

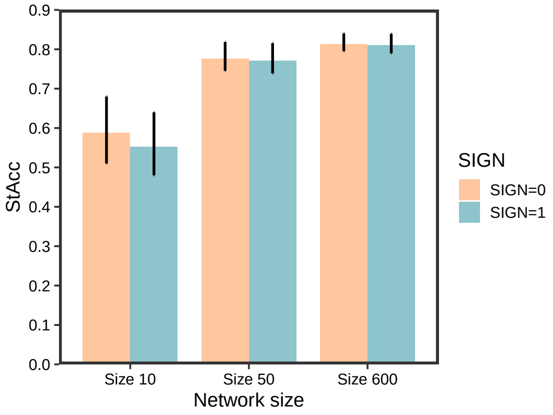

Figure S5 The StAcc value of LogBTF method on three types of simulated single-cell datasets with different sizes.

All experimental results, including AUROC, AUPR, StAcc, Recal, Pre, FPR and F-measure), are available in Table S3 - Table S6.

Table S3 The detailed experimental results on simulated single-cell datasets under SIGN=1.

Size	Dataset	Method	Mean AUC	DyAcc	AUROC	AUPR	StAcc	Recal	Pre	FPR	*F*-measure
10	Ecoli1	LogBTF	0.952	0.960	0.674	0.199	0.620	0.818	0.200	0.404	0.321
		SINCERITIES	–	–	0.309	0.005	0.580	0.000	0.000	0.383	0.000
	Ecoli2	LogBTF	0.994	0.990	0.489	0.141	0.520	0.500	0.125	0.477	0.200
		SINCERITIES	–	–	0.410	0.055	0.570	0.182	0.056	0.382	0.085
	Yeast1	LogBTF	0.950	0.995	0.490	0.063	0.480	0.571	0.075	0.527	0.133
		SINCERITIES	–	–	0.339	0.005	0.620	0.000	0.000	0.333	0.000
	Yeast2	LogBTF	0.926	0.935	0.509	0.222	0.500	0.765	0.220	0.554	0.342
		SINCERITIES	–	–	0.522	0.283	0.570	0.348	0.222	0.364	0.271
	Yeast3	LogBTF	0.853	0.945	0.626	0.335	0.640	0.611	0.275	0.354	0.379
		SINCERITIES	–	–	0.361	0.068	0.520	0.105	0.061	0.383	0.077
50	Ecoli1	LogBTF	0.990	0.991	0.527	0.032	0.759	0.259	0.024	0.230	0.044
		SINCERITIES	–	–	0.506	0.018	0.815	0.189	0.023	0.171	0.041
	Ecoli2	LogBTF	0.970	0.997	0.533	0.030	0.754	0.284	0.032	0.233	0.058
		SINCERITIES	–	–	0.447	0.008	0.836	0.027	0.006	0.139	0.010
	Yeast1	LogBTF	0.948	0.993	0.497	0.028	0.816	0.171	0.029	0.166	0.049
		SINCERITIES	–	–	0.452	0.009	0.822	0.044	0.008	0.156	0.013
	Yeast2	LogBTF	0.988	0.987	0.472	0.039	0.739	0.167	0.043	0.226	0.068
		SINCERITIES	–	–	0.468	0.034	0.798	0.082	0.031	0.157	0.045
	Yeast3	LogBTF	0.995	0.995	0.501	0.063	0.790	0.163	0.062	0.167	0.090
		SINCERITIES	–	–	0.475	0.036	0.806	0.093	0.042	0.145	0.058
100	Ecoli1	LogBTF	0.987	0.986	0.516	0.020	0.822	0.203	0.015	0.170	0.027
		SINCERITIES	–	–	0.451	0.004	0.855	0.055	0.004	0.136	0.008
	Ecoli2	LogBTF	0.993	0.989	0.509	0.019	0.794	0.211	0.012	0.199	0.022
		SINCERITIES	–	–	0.471	0.006	0.863	0.065	0.005	0.128	0.010
	Yeast1	LogBTF	0.990	0.988	0.504	0.023	0.839	0.153	0.016	0.150	0.029
		SINCERITIES	–	–	0.464	0.006	0.880	0.026	0.004	0.106	0.007
	Yeast2	LogBTF	0.992	0.989	0.485	0.029	0.805	0.140	0.029	0.171	0.048
		SINCERITIES	–	–	0.472	0.018	0.850	0.056	0.017	0.121	0.026
	Yeast3	LogBTF	0.994	0.993	0.471	0.034	0.790	0.114	0.033	0.175	0.051
		SINCERITIES	–	–	0.471	0.023	0.838	0.044	0.020	0.119	0.028

Table S4 The detailed experimental results on simulated single-cell datasets with size 10 under SIGN=0.

Size	Dataset	Method	AUROC	AUPR	StAcc	Recal	Pre	FPR	*F*-measure
10	*Ecoli*1	LogBTF	0.674	0.200	0.620	0.818	0.200	0.404	0.321
		SCODE	0.634	0.138	0.110	1.000	0.110	1.000	0.198
		GRISLI	0.410	0.096	–	–	–	–	–
		SINCERITIES	0.450	0.132	0.600	0.250	0.056	0.370	0.091
		GENIE3	0.469	0.416	0.580	0.636	0.156	0.427	0.250
		TREGRESS	0.541	0.519	0.620	0.818	0.200	0.404	0.321
		ARACNE	0.000	0.555	0.110	1.000	0.110	1.000	0.198
		CLR	0.000	0.555	0.110	1.000	0.110	1.000	0.198
	*Ecoli*2	LogBTF	0.565	0.233	0.550	0.600	0.188	0.459	0.286
		SCODE	0.451	0.126	0.150	1.000	0.150	1.000	0.261
		GRISLI	0.539	0.199	–	–	–	–	–
		SINCERITIES	0.480	0.120	0.590	0.308	0.111	0.368	0.163
		GENIE3	0.489	0.501	0.590	0.733	0.229	0.435	0.349
		TREGRESS	0.433	0.424	0.550	0.600	0.188	0.459	0.286
		ARACNE	0.000	0.575	0.150	1.000	0.150	1.000	0.261
		CLR	0.380	0.346	0.510	0.467	0.146	0.482	0.222
	*Yeast*1	LogBTF	0.574	0.162	0.510	0.700	0.132	0.511	0.222
		SCODE	0.489	0.115	0.100	1.000	0.100	1.000	0.182
		GRISLI	0.570	0.121	–	–	–	–	–
		SINCERITIES	0.483	0.090	0.650	0.300	0.097	0.311	0.146
		GENIE3	0.350	0.322	0.470	0.500	0.094	0.533	0.159
		TREGRESS	0.350	0.322	0.470	0.500	0.094	0.533	0.159
		ARACNE	0.111	0.556	0.200	1.000	0.111	0.889	0.200
		CLR	0.000	0.550	0.100	1.000	0.100	1.000	0.182
	*Yeast*2	LogBTF	0.603	0.351	0.580	0.840	0.356	0.507	0.500
		SCODE	0.490	0.236	0.250	1.000	0.250	1.000	0.400
		GRISLI	0.375	0.227	–	–	–	–	–
		SINCERITIES	0.517	0.264	0.570	0.348	0.222	0.364	0.271
		GENIE3	0.350	0.501	0.480	0.640	0.271	0.573	0.381
		TREGRESS	0.390	0.548	0.520	0.720	0.305	0.547	0.429
		ARACNE	0.294	0.586	0.450	0.840	0.292	0.680	0.433
		CLR	0.319	0.590	0.470	0.840	0.300	0.653	0.442
	*Yeast*3	LogBTF	0.695	0.464	0.680	0.682	0.375	0.321	0.484
		SCODE	0.507	0.223	0.220	1.000	0.220	1.000	0.361
		GRISLI	0.497	0.234	–	–	–	–	–
		SINCERITIES	0.406	0.155	0.540	0.190	0.121	0.367	0.148
		GENIE3	0.402	0.352	0.540	0.364	0.200	0.410	0.258
		TREGRESS	0.495	0.473	0.620	0.545	0.300	0.359	0.387
		ARACNE	0.363	0.317	0.500	0.318	0.167	0.449	0.219
		CLR	0.338	0.261	0.480	0.227	0.125	0.449	0.161

Table S5 The detailed experimental results on simulated single-cell datasets with size 50 under SIGN=0.

Size	Dataset	Method	AUROC	AUPR	StAcc	Recal	Pre	FPR	*F*-measure
50	*Ecoli*1	LogBTF	0.583	0.086	0.762	0.355	0.038	0.227	0.069
		SCODE	0.508	0.024	0.025	1.000	0.025	1.000	0.048
		GRISLI	0.512	0.024	–	–	–	–	–
		SINCERITIES	0.495	0.023	0.814	0.157	0.019	0.172	0.033
		GENIE3	0.427	0.074	0.750	0.113	0.012	0.233	0.022
		TREGRESS	0.490	0.161	0.758	0.274	0.029	0.230	0.053
		ARACNE	0.495	0.169	0.755	0.290	0.031	0.233	0.056
		CLR	0.470	0.143	0.745	0.242	0.025	0.242	0.045
	*Ecoli*2	LogBTF	0.603	0.065	0.760	0.415	0.058	0.228	0.102
		SCODE	0.583	0.050	0.033	1.000	0.033	1.000	0.064
		GRISLI	0.526	0.041	–	–	–	–	–
		SINCERITIES	0.467	0.023	0.838	0.065	0.015	0.138	0.024
		GENIE3	0.442	0.104	0.744	0.159	0.022	0.237	0.039
		TREGRESS	0.479	0.158	0.746	0.256	0.035	0.237	0.062
		ARACNE	0.476	0.151	0.748	0.244	0.034	0.235	0.060
		CLR	0.432	0.091	0.741	0.134	0.019	0.239	0.033
	*Yeast*1	LogBTF	0.539	0.053	0.818	0.247	0.046	0.163	0.077
		SCODE	0.520	0.039	0.031	1.000	0.031	1.000	0.060
		GRISLI	0.493	0.028	–	–	–	–	–
		SINCERITIES	0.459	0.019	0.822	0.058	0.010	0.156	0.018
		GENIE3	0.476	0.098	0.812	0.143	0.027	0.167	0.045
		TREGRESS	0.504	0.135	0.815	0.208	0.038	0.165	0.065
		ARACNE	0.305	0.274	0.408	0.506	0.026	0.596	0.050
		CLR	0.500	0.135	0.809	0.208	0.037	0.172	0.063
	*Yeast*2	LogBTF	0.518	0.072	0.745	0.250	0.072	0.221	0.112
		SCODE	0.493	0.069	0.064	1.000	0.064	1.000	0.120
		GRISLI	0.533	0.068	–	–	–	–	–
		SINCERITIES	0.454	0.040	0.797	0.056	0.021	0.159	0.031
		GENIE3	0.490	0.189	0.746	0.256	0.074	0.221	0.114
		TREGRESS	0.483	0.181	0.743	0.244	0.070	0.223	0.108
		ARACNE	0.368	0.291	0.494	0.488	0.062	0.505	0.110
		CLR	0.479	0.183	0.733	0.250	0.068	0.234	0.107
	*Yeast*3	LogBTF	0.530	0.097	0.795	0.225	0.094	0.162	0.132
		SCODE	0.423	0.059	0.069	1.000	0.069	1.000	0.129
		GRISLI	0.503	0.069	–	–	–	–	–
		SINCERITIES	0.463	0.044	0.803	0.058	0.025	0.148	0.035
		GENIE3	0.489	0.151	0.788	0.173	0.072	0.166	0.102
		TREGRESS	0.497	0.163	0.790	0.191	0.079	0.165	0.112
		ARACNE	0.446	0.240	0.633	0.364	0.072	0.347	0.121
		CLR	0.469	0.124	0.780	0.133	0.055	0.171	0.077

Table S6 The detailed experimental results on simulated single-cell datasets with size 100 under SIGN=0.

Size	Dataset	Method	AUROC	AUPR	StAcc	Recal	Pre	FPR	F-measure
100		LogBTF	0.523	0.027	0.822	0.216	0.016	0.170	0.029
		SCODE	0.539	0.013	0.013	1.000	0.013	1.000	0.025
		GRISLI	0.515	0.014	–	–	–	–	–
	*Ecoli*1	SINCERITIES	0.442	0.006	0.855	0.019	0.001	0.136	0.003
		GENIE3	0.498	0.112	0.822	0.200	0.015	0.170	0.027
		TREGRESS	0.535	0.159	0.824	0.288	0.021	0.170	0.039
		ARACNE	0.510	0.137	0.810	0.248	0.017	0.183	0.032
		CLR	0.517	0.138	0.821	0.248	0.018	0.172	0.033
		LogBTF	0.546	0.029	0.795	0.277	0.017	0.199	0.031
		SCODE	0.539	0.013	0.013	1.000	0.013	1.000	0.025
		GRISLI	0.507	0.012	–	–	–	–	–
	*Ecoli*2	SINCERITIES	0.468	0.008	0.863	0.057	0.005	0.128	0.009
		GENIE3	0.433	0.050	0.791	0.084	0.005	0.201	0.009
		TREGRESS	0.496	0.134	0.791	0.244	0.014	0.202	0.027
		ARACNE	0.500	0.172	0.753	0.319	0.016	0.242	0.030
		CLR	0.496	0.133	0.791	0.244	0.014	0.203	0.027
		LogBTF	0.528	0.033	0.840	0.199	0.022	0.149	0.040
		SCODE	0.541	0.018	0.017	1.000	0.017	1.000	0.033
		GRISLI	0.485	0.016	–	–	–	–	–
	*Yeast*1	SINCERITIES	0.455	0.008	0.880	0.007	0.001	0.107	0.002
		GENIE3	0.505	0.110	0.840	0.187	0.021	0.149	0.037
		TREGRESS	0.492	0.094	0.839	0.157	0.017	0.150	0.031
		ARACNE	0.379	0.287	0.490	0.548	0.018	0.511	0.034
		CLR	0.488	0.091	0.837	0.151	0.017	0.151	0.030
		LogBTF	0.531	0.056	0.809	0.229	0.052	0.168	0.085
		SCODE	0.545	0.051	0.039	1.000	0.039	1.000	0.075
		GRISLI	0.465	0.037	–	–	–	–	–
	*Yeast*2	SINCERITIES	0.456	0.019	0.848	0.015	0.004	0.122	0.007
		GENIE3	0.478	0.110	0.803	0.152	0.035	0.171	0.056
		TREGRESS	0.479	0.113	0.802	0.157	0.036	0.172	0.058
		ARACNE	0.349	0.289	0.461	0.522	0.038	0.541	0.070
		CLR	0.474	0.109	0.798	0.152	0.034	0.176	0.055
		LogBTF	0.514	0.063	0.795	0.200	0.064	0.170	0.097
		SCODE	0.482	0.052	0.055	1.000	0.055	1.000	0.104
		GRISLI	0.517	0.061	–	–	–	–	–
	*Yeast*3	SINCERITIES	0.468	0.033	0.837	0.035	0.016	0.119	0.022
		GENIE3	0.466	0.109	0.787	0.129	0.041	0.174	0.063
		TREGRESS	0.498	0.156	0.794	0.203	0.065	0.172	0.098
		ARACNE	0.358	0.296	0.475	0.512	0.054	0.527	0.097
		CLR	0.489	0.145	0.789	0.187	0.058	0.176	0.089

9. Results on synthetic bulk gene expression data by GWN

9.1. Case 1: SIGN = 1

In this experiment, we also use the LOOCV method (James et al., 2013) to select the optimal tuning parameter and predict the expression state of each gene on each dataset to investigate the performance of LogBTF in terms of predictive AUC value. Figure S6 shows the experiment results. Figure S6(a) shows that the means of the AUC value and DyAcc value on 15 datasets are both larger than 0.98. Considering that the SINCERITIES method is also functional in evaluating the inferred network from the aspect of activating and inhibitive regulations between regulator and target genes, we compare the StAcc and AUROC indexes of LogBTF with SINCERITIES.

From Figure S6(b), one can see that the StAcc values of LogBTF are larger than SINCERITIES in most cases when the network size is small, but the mean StAcc value is smaller than SINCERITIES. While as the network size increases, our LogBTF method shows strong superiority when compared with SINCERITIES, both from single experimental results and mean value. Furthermore, the larger the number of nodes in the network, the higher the StAcc value of our method. Figure S6(c) shows the results of the AUROC value comparing the LogBTF with the SINCERITIES method on three types of datasets of different sizes. We can see that our proposed LogBTF method performs better than SINCERITIES for all network sizes. Also, there seems to be a trend that when the number of nodes/genes in the network increases, the difference between these two methods gradually decreases (under the premise that LogBTF is better than SINCERITIES).

9.2. Case 2: SIGN = 0

When omitting the activation or inhibition functions, we only study the regulatory relationships and the regulator/target roles among genes, i.e., SIGN=0. In this case, the signature of regression or correlation coefficient does not work, which means that all coefficients can be taken as the measure like weights. Thus these six network inference methods have a standard benchmark for making comparisons. For more reliable performance validation, we further compare LogBTF with five methods, including SINCERITIES that we just discussed, GENIE3, TIGRESS, ARACNE

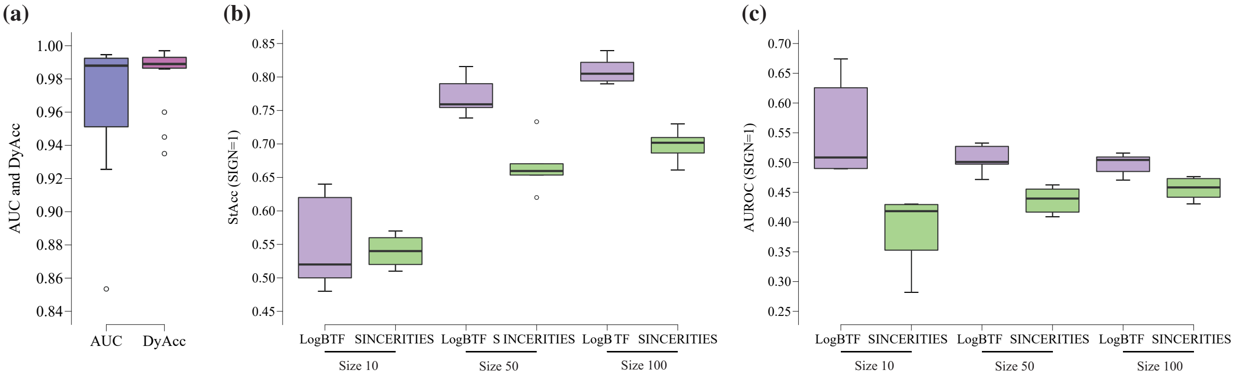

Figure S6 The prediction results of LogBTF method on DREAM3 change dataset in the case of SNGN=1. (a) The AUC and DyAcc values on 15 datasets in DREAM3 change. (b) The StAcc and (c) AUROC comparison of LogBTF and SINCERITIES methods on three types of datasets of different sizes.

and CLR. As we all know, the inferred interactions/links of four methods, except SINCERITIES, require a cut-off value to truncate the weight or correlation coefficient to ensure comparability with our proposed method. In the experiments, we determine the cut-off threshold value corresponding to the number κ of edges predicted by our method by arranging all edges' weights or correlations in descending order. The comparing results of StAcc, AUROC and Pre indexes on all available datasets are shown in Figure S7.

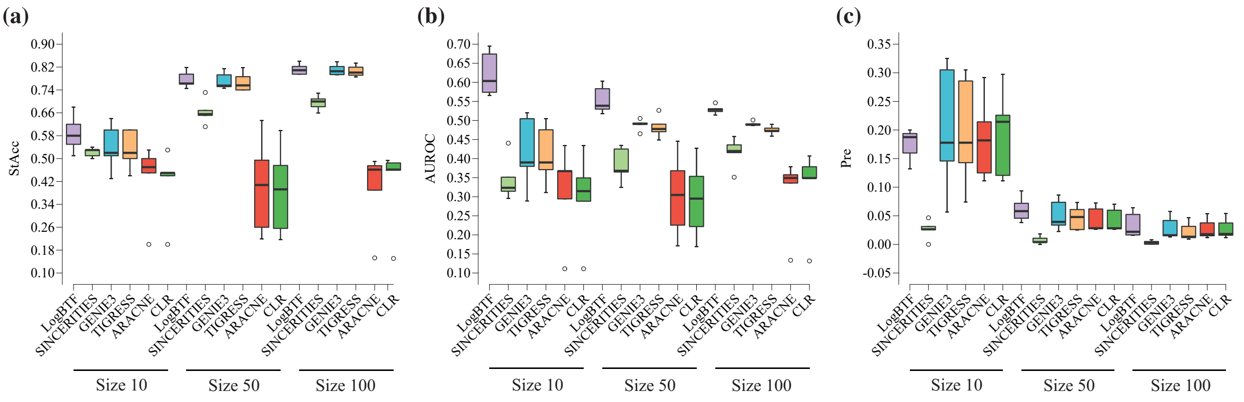

Figure S7 The comparing results of LogBTF with the other five methods on three type datasets with different gene sizes from DREAM3 change in the case of SNGN=0. (a) The StAcc values. (b) The AUROC value. (c) Pre value.

Figure S7(a) shows the StAcc values among all methods, where the LogBTF method achieves the best results regardless of the network size of the dataset. In particular, when the size of network nodes is relatively large (such as size 100), our method has the largest mean StAcc and the smallest standard deviation in five datasets experiments. Figure S7(b) displays that our proposed method is significantly superior to the other five comparable methods in terms of AUROC evaluation, regardless of the size of the network. Figure S7(c) gives the Pre values comparing the inferred network relationship with sourced gold standards. In the five datasets with 10 genes, the LogBTF method achieves worse Pre values and whose mean Pre value is also lower than the CLR method, but it should be noted that whose standard deviation is very small compared with GENIE3, TIGRESS, ARACNE and CLR methods. And, this situation is also comfortable for the case of size 50 and size 100. As for SINCERITIES, it obtains the smallest standard deviation while its Pre values are the worst among all methods on all 15 datasets. All experimental results, including other estimation criteria (such as Recal, FPR and F-measure), are available in Table S7. They reflect the stability and applicability of the LogBTF method for large-scale networks.

Table S7 The detailed experimental results on simulated “bulk” gene expression datasets.

# of genes	Dataset	Method	# of min genes	# of max genes	# of regulatory relationships	Mean AUC	DyAcc	AUROC	AUPR	StAcc	Recal	Pre	FPR	*F*-measure	AUROC	AUPR	StAcc	Recal	Pre	FPR	*F*-measure
								Activate or inhibit function (SIGN = 1)							No activate or inhibit function (SIGN = 0)						
10	Ecoli1	LogBTF	3	6	45	0.952	0.960	0.674	0.199	0.620	0.818	0.200	0.404	0.321	0.674	0.200	0.620	0.818	0.200	0.404	0.321
		SINCER–	2	6	43			0.282	0.005	0.510	0.000	0.000	0.457	0.000	0.440	0.084	0.530	0.250	0.047	0.446	0.078
		GENIE3			45										0.505	0.468	0.600	0.727	0.178	0.416	0.286
		TIGRESS			45										0.505	0.468	0.600	0.727	0.178	0.416	0.286
		ARACNE			45										0.368	0.401	0.470	0.636	0.125	0.551	0.209
		CLR			45										0.349	0.399	0.450	0.636	0.121	0.573	0.203
	Ecoli2	LogBTF	4	7	48	0.994	0.990	0.489	0.141	0.520	0.500	0.125	0.477	0.200	0.565	0.233	0.550	0.600	0.188	0.459	0.286
		SINCER–	2	6	39			0.353	0.053	0.520	0.154	0.051	0.425	0.077	0.314	0.068	0.510	0.083	0.026	0.432	0.039
		GENIE3			48										0.380	0.346	0.510	0.467	0.146	0.482	0.222
		TIGRESS			48										0.371	0.345	0.500	0.467	0.143	0.494	0.219
		ARACNE			48										0.434	0.522	0.530	0.800	0.214	0.518	0.338
		CLR			48										0.434	0.522	0.530	0.800	0.214	0.518	0.338
	Yeast1	LogBTF	2	8	53	0.950	0.995	0.490	0.063	0.480	0.571	0.075	0.527	0.133	0.574	0.162	0.510	0.700	0.132	0.511	0.222
		SINCER–	3	5	39			0.418	0.055	0.570	0.300	0.077	0.400	0.122	0.296	0.035	0.540	0.000	0.000	0.419	0.000
		GENIE3			53										0.289	0.213	0.430	0.300	0.057	0.556	0.095
		TIGRESS			53										0.311	0.267	0.440	0.400	0.074	0.556	0.125
		ARACNE			53										0.111	0.556	0.200	1.000	0.111	0.889	0.200
		CLR			53										0.111	0.556	0.200	1.000	0.111	0.889	0.200
	Yeast2	LogBTF	4	7	59	0.926	0.935	0.509	0.222	0.500	0.765	0.220	0.554	0.342	0.603	0.351	0.580	0.840	0.356	0.507	0.500
		SINCER–	2	6	38			0.430	0.121	0.540	0.278	0.132	0.402	0.179	0.323	0.081	0.500	0.071	0.026	0.430	0.038
		GENIE3			59										0.390	0.548	0.520	0.720	0.305	0.547	0.429
		TIGRESS			59										0.390	0.548	0.520	0.720	0.305	0.547	0.429
		ARACNE			59										0.294	0.586	0.450	0.840	0.292	0.680	0.433
		CLR			59										0.288	0.604	0.450	0.880	0.297	0.693	0.444
	Yeast3	LogBTF	2	5	40	0.853	0.945	0.626	0.335	0.640	0.611	0.275	0.354	0.379	0.695	0.464	0.680	0.682	0.375	0.321	0.484
		SINCER–	2	5	32			0.429	0.112	0.560	0.200	0.125	0.350	0.154	0.351	0.098	0.530	0.059	0.031	0.373	0.041
		GENIE3			40										0.520	0.503	0.640	0.591	0.325	0.346	0.419
		TIGRESS			40										0.476	0.466	0.600	0.545	0.286	0.385	0.375
		ARACNE			40										0.367	0.343	0.500	0.364	0.182	0.462	0.242
		CLR			40										0.315	0.471	0.440	0.636	0.226	0.615	0.333
50	Ecoli1	LogBTF	3	23	576	0.990	0.991	0.527	0.032	0.759	0.259	0.024	0.230	0.044	0.583	0.086	0.762	0.355	0.038	0.227	0.069
		SINCER–	5	26	920			0.462	0.020	0.620	0.292	0.023	0.370	0.042	0.325	0.010	0.612	0.000	0.000	0.376	0.069
		GENIE3			576										0.465	0.126	0.755	0.210	0.023	0.231	0.000
		TIGRESS			576										0.478	0.143	0.756	0.242	0.026	0.231	0.047
		ARACNE			576										0.226	0.435	0.260	0.839	0.027	0.755	0.053
		CLR			576										0.222	0.435	0.256	0.839	0.027	0.759	0.053
	Ecoli2	LogBTF	4	20	585	0.970	0.997	0.533	0.030	0.754	0.284	0.032	0.233	0.058	0.603	0.065	0.760	0.415	0.058	0.228	0.102
		SINCER–	3	25	789			0.417	0.011	0.670	0.157	0.010	0.319	0.019	0.365	0.010	0.668	0.065	0.004	0.320	0.007
		GENIE3			585										0.491	0.172	0.752	0.280	0.039	0.232	0.069
		TIGRESS			585										0.449	0.117	0.740	0.183	0.025	0.241	0.044
		ARACNE			585										0.171	0.361	0.219	0.683	0.028	0.797	0.054
		CLR			585										0.169	0.361	0.217	0.683	0.028	0.799	0.054
	Yeast1	LogBTF	2	15	415	0.948	0.993	0.497	0.028	0.816	0.171	0.029	0.166	0.049	0.539	0.053	0.818	0.247	0.046	0.163	0.077
		SINCER–	4	25	836			0.455	0.016	0.654	0.250	0.018	0.336	0.033	0.434	0.025	0.651	0.167	0.011	0.338	0.020
		GENIE3			415										0.493	0.120	0.814	0.182	0.034	0.165	0.057
		TIGRESS			415										0.526	0.165	0.818	0.260	0.048	0.164	0.081
		ARACNE			415										0.305	0.274	0.408	0.506	0.026	0.596	0.050
		CLR			415										0.295	0.280	0.392	0.519	0.026	0.612	0.050
	Yeast2	LogBTF	4	16	557	0.988	0.987	0.472	0.039	0.739	0.167	0.043	0.226	0.068	0.518	0.072	0.745	0.250	0.072	0.221	0.112
		SINCER–	3	23	748			0.409	0.024	0.660	0.107	0.019	0.310	0.032	0.368	0.026	0.655	0.025	0.004	0.313	0.007
		GENIE3			557										0.490	0.189	0.746	0.256	0.074	0.221	0.114
		TIGRESS			557										0.470	0.162	0.740	0.213	0.061	0.224	0.095
		ARACNE			557										0.368	0.291	0.494	0.488	0.062	0.505	0.110
		CLR			557										0.353	0.290	0.476	0.488	0.060	0.525	0.106
	Yeast3	LogBTF	3	18	417	0.995	0.995	0.501	0.063	0.790	0.163	0.062	0.167	0.090	0.530	0.097	0.795	0.225	0.094	0.162	0.132
		SINCER–	5	16	546			0.440	0.027	0.733	0.099	0.027	0.226	0.043	0.425	0.038	0.731	0.068	0.018	0.228	0.029
		GENIE3			417										0.505	0.175	0.793	0.208	0.086	0.164	0.122
		TIGRESS			417										0.490	0.155	0.786	0.179	0.073	0.168	0.104
		ARACNE			417										0.446	0.240	0.633	0.364	0.072	0.347	0.121
		CLR			417										0.427	0.253	0.598	0.393	0.070	0.387	0.119

# of genes	Dataset	Method	# of min genes	# of max genes	# of regulatory relationships	Mean AUC	DyAcc	AUROC	AUPR	StAcc	Recal	Pre	FPR	*F*-measure	AUROC	AUPR	StAcc	Recal	Pre	FPR	*F*-measure
								Activate or inhibit function (SIGN = 1)							No activate or inhibit function (SIGN = 0)						
100	Ecoli1	LogBTF	2	43	1708	0.987	0.986	0.516	0.020	0.822	0.203	0.015	0.170	0.027	0.523	0.027	0.822	0.216	0.016	0.170	0.029
		SINCER−	4	49	2833			0.476	0.005	0.710	0.118	0.004	0.285	0.008	0.458	0.005	0.709	0.012	0.000	0.286	0.001
		GENIE3			1708										0.501	0.117	0.822	0.208	0.015	0.170	0.028
		TIGRESS			1708										0.490	0.104	0.820	0.184	0.013	0.172	0.025
		ARACNE			1708										0.336	0.381	0.390	0.744	0.015	0.615	0.030
		CLR			1708										0.407	0.338	0.494	0.656	0.016	0.509	0.031
	Ecoli2	LogBTF	4	34	1996	0.993	0.989	0.509	0.019	0.794	0.211	0.012	0.199	0.022	0.546	0.029	0.795	0.277	0.017	0.199	0.031
		SINCER−	4	50	3335			0.431	0.005	0.661	0.200	0.005	0.335	0.011	0.351	0.004	0.659	0.014	0.000	0.336	0.001
		GENIE3			1996										0.488	0.120	0.794	0.218	0.013	0.199	0.025
		TIGRESS			1996										0.480	0.112	0.792	0.202	0.012	0.201	0.023
		ARACNE			1996										0.133	0.431	0.153	0.849	0.012	0.856	0.023
		CLR			1996										0.132	0.431	0.151	0.849	0.012	0.858	0.023
	Yeast1	LogBTF	5	29	1497	0.990	0.988	0.504	0.023	0.839	0.153	0.016	0.150	0.029	0.528	0.033	0.840	0.199	0.022	0.149	0.040
		SINCER−	3	36	2602			0.458	0.008	0.730	0.150	0.008	0.262	0.015	0.420	0.008	0.729	0.063	0.003	0.263	0.006
		GENIE3			1497										0.487	0.087	0.839	0.145	0.016	0.150	0.029
		TIGRESS			1497										0.459	0.054	0.834	0.084	0.009	0.154	0.017
		ARACNE			1497										0.379	0.287	0.490	0.548	0.018	0.511	0.034
		CLR			1497										0.379	0.296	0.485	0.566	0.018	0.517	0.035
	Yeast2	LogBTF	4	35	1700	0.992	0.989	0.485	0.029	0.805	0.140	0.029	0.171	0.048	0.531	0.056	0.809	0.229	0.052	0.168	0.085
		SINCER−	4	46	2743			0.442	0.017	0.702	0.132	0.016	0.279	0.028	0.416	0.019	0.700	0.072	0.008	0.281	0.014
		GENIE3			1700										0.491	0.128	0.805	0.183	0.042	0.169	0.068
		TIGRESS			1700										0.471	0.102	0.801	0.139	0.032	0.173	0.051
		ARACNE			1700										0.349	0.289	0.461	0.522	0.038	0.541	0.070
		CLR			1700										0.349	0.288	0.462	0.519	0.037	0.541	0.070
	Yeast3	LogBTF	4	38	1718	0.994	0.993	0.471	0.034	0.790	0.114	0.033	0.175	0.051	0.514	0.063	0.795	0.200	0.064	0.170	0.097
		SINCER−	3	42	2789			0.473	0.030	0.687	0.135	0.023	0.286	0.039	0.436	0.024	0.682	0.033	0.005	0.290	0.009
		GENIE3			1718										0.489	0.141	0.793	0.180	0.058	0.171	0.087
		TIGRESS			1718										0.473	0.121	0.786	0.149	0.047	0.177	0.071
		ARACNE			1718										0.358	0.296	0.475	0.512	0.054	0.527	0.097
		CLR			1718										0.349	0.307	0.459	0.534	0.054	0.545	0.098

10. Results on real single-cell data

10.1. Matsumoto and hHEP data

Figure S8 shows the comparison results of AUROC and computational time of LogBTF with the other seven methods on hHEP dataset, where LogBTF method behaves with higher AUROC values than those of the other seven methods and SINCERITIES shows a second-best AUROC.

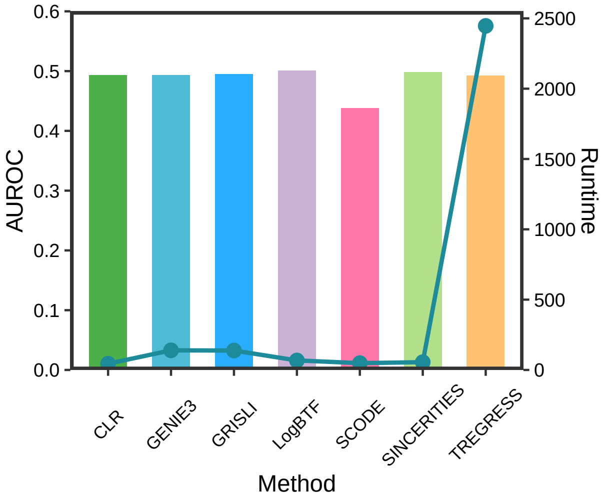

Figure S8 The performance comparison among different methods.

The detailed comparison results of LogBTF with the other seven methods on Matsumoto and hHEP dataset are shown in Table S8. Regarding StAcc, SINCERITIES performs best in both Matsumoto and specific hHEP datasets. All other methods are worse than those two methods in StAcc. For GRISLI, Aubin-Frankowski & Vert (2020) has stressed that the performance of GRISLI method is impacted by two main parameters and the choice of the parameters is a difficult question. Considering that hHEP includes nearly one thousand genes, here we take $R = L = 10$ in its source code for saving runtimes. As for GNIPLR, it prints "ConvergenceWarning: Objective did not converge" on hHEP dataset, so the results can not be collected.

Table S8 The performance comparison of inferring GRNs on two real scRNA-seq datasets under SIGN=0, where the '–' represents that the evaluation values are not available using the other inferring methods. All experiments are conducted on a workstation with two Xeon Gold 6226R CPUs and 256G of RAM.

Dataset	Method	Mean AUC	DyAcc	AUROC	AUPR	StAcc	Recal	Pre	FPR	*F*-measure	Time (min)
Matsumoto scRNA-seq data (373*100)	LogBTF	0.577	0.697	0.507	0.072	0.755	0.218	0.070	0.207	0.106	11.741
	GRISLI	–	–	0.502	0.426	–	–	–	–	–	14.256
	SCODE	–	–	0.478	0.063	0.067	1.000	0.067	1.000	0.125	0.249
	SINCERITIES	–	–	0.491	0.064	0.778	0.156	0.059	0.178	0.086	7.502
	GENIE3	–	–	0.483	0.170	0.755	0.218	0.070	0.207	0.106	3.911
	TREGRESS	–	–	0.492	0.186	0.754	0.245	0.077	0.210	0.117	150.604
	ARACNE	–	–	0.000	0.533	0.067	1.000	0.067	1.000	0.125	0.049
	CLR	–	–	0.481	0.167	0.754	0.213	0.068	0.207	0.104	0.048
	GNIPLR	–	–	0.498	0.191	0.759	0.251	0.081	0.204	0.122	10.868
Specific hHEP scRNA-seq data (425*948)	LogBTF	0.708	0.731	0.501	0.012	0.950	0.043	0.012	0.040	0.019	25.174
	GRISLI	–	–	0.495	0.013	–	–	–	–	–	95.494
	SCODE	–	–	0.438	0.009	0.011	1.000	0.011	1.000	0.022	5.93
	SINCERITIES	–	–	0.498	0.010	0.959	0.026	0.009	0.031	0.014	12.893
	GENIE3	–	–	0.493	0.023	0.950	0.028	0.008	0.040	0.012	97.019
	TIGRESS	–	–	0.492	0.022	0.948	0.027	0.007	0.041	0.011	2403.995
	ARACNE	–	–	0.000	0.506	0.011	1.000	0.011	1.000	0.022	1.934
	CLR	–	–	0.493	0.023	0.949	0.027	0.008	0.040	0.012	1.896
	GNIPLR	–	–	–	–	–	–	–	–	–	–

In particular, ARACNE and CLR are two inference algorithms based on spearman estimator/correlation, but the inferred results on these two large real networks are very different. Thus, ARACNE seems not appropriate in the case of scRNA-seq data with pseudo-time, as mentioned in prior work that it is not for the time series data (Cantone et al., 2009). In particular, we note that GRISLI and TIGRESS are not suitable for large-scale network inference analysis due to the expensive computational time, though they can also obtain satisfactory inference results. Clearly, our LogBTF method performs better on both Matsumoto and hHEP scRNA-seq datasets.

10.2. LMPP data

For the LMPP scRNA-seq dataset, LogBTF method infers 306 regulatory relationships, shown in Figure S9.

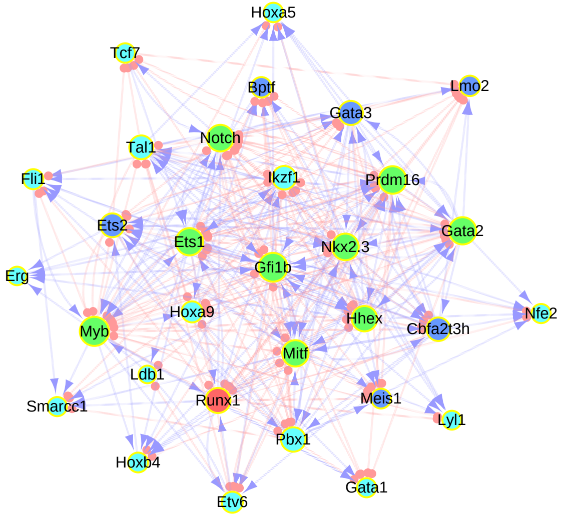

Figure S9 The network topology of reconstructed GRN that reflects the differentiation of HSCs into LMPP, where the node's size is proportional to its degree (sum of out-degree and in-degree). The genes with the top 15 activations are colored in blue, the genes with the top 10 inhibitions are colored in red, and the genes with both high activation and high inhibitions regulation relationships are marked in green (9 in total).

10.3. NOA enrichment results

The functional enrichment analysis results (P-value < 0.05) enriched by NOA are listed in Table S9.

11. Discussion

Many existing gene regulatory network inference algorithms may suffer from the over-fitting problem, which means that the inferred in-degree tends to be larger than the real in-degree of the nodes (Liu et al., 2021). However, our LogBTF method embeds regularized logistic regression into the Boolean threshold network model to automatically make regulatory link selection when fitting the input gene using input gene expression data. LogBTF takes regulatory relationship inference for each gene as an embedded feature selection process and successfully handles the over-fitting problem.

Most recently developed inference methods that need to determine the maximum in-degree of each gene considering the sparsity of networks. For example, Liu et al. fixed the maximum in-degree as five for each gene when applying a Boolean network model to real-world gene regulatory network inference problems (Liu et al., 2021). Barman and Kwon set the maximum number of regulatory genes as three-fifths of the total number of all genes for the Boolean network inference to save the search cost of the neural network (Barman & Kwon, 2020). Barman and Kwon also constrained the maximum in-degree for the target gene to reduce the search space of the genetic algorithm when conducting the Boolean network inference problems (Barman & Kwon, 2018). In contrast, our proposed LogBTF

Table S9 The functional enrichment analysis results enriched by NOA (Wang et al., 2011).

GO: term	*P*-value	Corrected *P*-value	R	T	G	O	Term name
GO:0048518	2.00E-06	3.50E-04	379	177	91	62	positive regulation of biological process
GO:0010468	1.50E-05	0.0026	379	177	325	166	regulation of gene expression
GO:0010556	1.50E-05	0.0026	379	177	325	166	regulation of macromolecule biosynthetic process
GO:0019219	1.50E-05	0.0026	379	177	325	166	regulation of nucleobase, nucleoside, nucleotide and nucleic acid metabolic process
GO:0019222	1.50E-05	0.0026	379	177	325	166	regulation of metabolic process
GO:0031323	1.50E-05	0.0026	379	177	325	166	regulation of cellular metabolic process
GO:0031326	1.50E-05	0.0026	379	177	325	166	regulation of cellular biosynthetic process
GO:0045449	1.50E-05	0.0026	379	177	325	166	regulation of transcription
GO:0051171	1.50E-05	0.0026	379	177	325	166	regulation of nitrogen compound metabolic process
GO:0060255	1.50E-05	0.0026	379	177	325	166	regulation of macromolecule metabolic process
GO:0080090	1.50E-05	0.0026	379	177	325	166	regulation of primary metabolic process
GO:0009889	1.50E-05	0.0026	379	177	325	166	regulation of biosynthetic process
GO:0048513	5.50E-05	0.0094	379	177	91	59	organ development
GO:0048522	5.80E-05	0.0099	379	177	78	52	positive regulation of cellular process
GO:0010557	7.50E-04	0.1291	379	177	55	37	positive regulation of macromolecule biosynthetic process
GO:0010604	7.50E-04	0.1291	379	177	55	37	positive regulation of macromolecule metabolic process
GO:0010628	7.50E-04	0.1291	379	177	55	37	positive regulation of gene expression
GO:0031325	7.50E-04	0.1291	379	177	55	37	positive regulation of cellular metabolic process
GO:0031328	7.50E-04	0.1291	379	177	55	37	positive regulation of cellular biosynthetic process
GO:0045935	7.50E-04	0.1291	379	177	55	37	positive regulation of nucleobase, nucleoside, nucleotide and nucleic acid metabolic process
GO:0045941	7.50E-04	0.1291	379	177	55	37	positive regulation of transcription
GO:0051173	7.50E-04	0.1291	379	177	55	37	positive regulation of nitrogen compound metabolic process
GO:0009891	7.50E-04	0.1291	379	177	55	37	positive regulation of biosynthetic process
GO:0009893	7.50E-04	0.1291	379	177	55	37	positive regulation of metabolic process
GO:0034641	0.0016	0.2842	379	177	276	142	cellular nitrogen compound metabolic process
GO:0034645	0.0016	0.2842	379	177	276	142	cellular macromolecule biosynthetic process
GO:0043170	0.0016	0.2842	379	177	276	142	macromolecule metabolic process
GO:0044237	0.0016	0.2842	379	177	276	142	cellular metabolic process
GO:0044238	0.0016	0.2842	379	177	276	142	primary metabolic process
GO:0044249	0.0016	0.2842	379	177	276	142	cellular biosynthetic process
GO:0044260	0.0016	0.2842	379	177	276	142	cellular macromolecule metabolic process
GO:0006139	0.0016	0.2842	379	177	276	142	nucleobase, nucleoside, nucleotide and nucleic acid metabolic process
GO:0006350	0.0016	0.2842	379	177	276	142	transcription
GO:0006807	0.0016	0.2842	379	177	276	142	nitrogen compound metabolic process
GO:0008152	0.0016	0.2842	379	177	276	142	metabolic process
GO:0090304	0.0016	0.2842	379	177	276	142	nucleic acid metabolic process
GO:0009058	0.0016	0.2842	379	177	276	142	biosynthetic process
GO:0009059	0.0016	0.2842	379	177	276	142	macromolecule biosynthetic process
GO:0006357	0.0019	0.3249	379	177	91	55	regulation of transcription from RNA polymerase II promoter
GO:0051239	0.0023	0.4083	379	177	78	48	regulation of multicellular organismal process
GO:0045893	0.0033	0.5708	379	177	36	25	positive regulation of transcription, DNA-dependent
GO:0045944	0.0033	0.5708	379	177	36	25	positive regulation of transcription from RNA polymerase II promoter
GO:0051254	0.0033	0.5708	379	177	36	25	positive regulation of RNA metabolic process
GO:0032502	0.0049	0.848	379	177	210	111	developmental process
GO:0051094	0.0055	0.9563	379	177	10	9	positive regulation of developmental process
GO:0048568	0.008	1	379	177	15	12	embryonic organ development
GO:0048869	0.0092	1	379	177	66	40	cellular developmental process
GO:0022603	0.0099	1	379	177	6	6	regulation of anatomical structure morphogenesis
GO:0045765	0.0099	1	379	177	6	6	regulation of angiogenesis
GO:0050793	0.0185	1	379	177	66	39	regulation of developmental process
GO:0030154	0.0232	1	379	177	55	33	cell differentiation

method does not need to set the maximum in-degree of each target node in advance when inferring gene regulatory network using the Boolean network model. LogBTF method automatically determines the number of regulatory genes according to the best-found solution to achieve a sparse regulatory network and perform the four levels (I, II, III and IV), as discussed in our Introduction section, of gene regulatory network inference problems.

In addition, the LogBTF method is an interpretable network inference method, where the regulatory rules are represented in Boolean threshold functions. Namely, for the normalized coefficients $\hat{\boldsymbol{\theta}}_i$, the ratio of the number of zero elements to the number of all components determines the sparsity of the inferred regulatory relationship among all genes, the value of no-zero element (large or small) determines the regulatory strength (strong or weak) between the regulator genes and the target gene, and the sign of non-zero element (positive/negative) determines whether the regulation is activated or inhibited.

References

Aubin-Frankowski, P.-C., & Vert, J.-P. (2020). Gene regulation inference from single-cell RNA-seq data with linear differential equations and velocity inference. *Bioinformatics*, *36*, 4774–4780. https://doi.org/10.1093/bioinformatics/btaa576.

Barman, S., & Kwon, Y.-K. (2018). A Boolean network inference from time-series gene expression data using a genetic algorithm. *Bioinformatics*, *34*, i927–i933. https://doi.org/10.1093/bioinformatics/bty584.

Barman, S., & Kwon, Y.-K. (2020). A neuro-evolution approach to infer a Boolean network from time-series gene expressions. *Bioinformatics*, *36*, i762–i769. https://doi.org/10.1093/bioinformatics/btaa840.

Bradley, A. P. (1997). The use of the area under the ROC curve in the evaluation of machine learning algorithms. *Pattern Recognition*, *30*, 1145–1159. https://doi.org/10.1016/S0031-3203(96)00142-2.

Camp, J. G., Sekine, K., Gerber, T., Loeffler-Wirth, H., Binder, H., Gac, M., Kanton, S., Kageyama, J., Damm, G., Seehofer, D. et al. (2017). Multilineage communication regulates human liver bud development from pluripotency. *Nature*, *546*, 533–538. https://doi.org/10.1038/nature22796.

Cantone, I., Marucci, L., Iorio, F., Ricci, M. A., Belcastro, V., Bansal, M., Santini, S., Di Bernardo, M., Di Bernardo, D., & Cosma, M. P. (2009). A yeast synthetic network for in vivo assessment of reverse-engineering and modeling approaches. *Cell*, *137*, 172–181. https://doi.org/10.1016/j.cell.2009.01.055.

Chen, S., & Mar, J. C. (2018). Evaluating methods of inferring gene regulatory networks highlights their lack of performance for single cell gene expression data. *BMC Bioinformatics*, *19*, 1–21. https://doi.org/10.1186/s12859-018-2217-z.

Dibaeinia, P., & Sinha, S. (2020). SERGIO: A single-cell expression simulator guided by gene regulatory networks. *Cell Systems*, *11*, 252–271. https://doi.org/10.1016/j.cels.2020.08.003.

Faith, J. J., Hayete, B., Thaden, J. T., Mogno, I., Wierzbowski, J., Cottarel, G., Kasif, S., Collins, J. J., & Gardner, T. S. (2007). Large-scale mapping and validation of Escherichia coli transcriptional regulation from a compendium of expression profiles. *PLoS Biology*, *5*, e8. https://doi.org/10.1371/journal.pbio.0050008.

Hamey, F. K., Nestorowa, S., Kinston, S. J., Kent, D. G., Wilson, N. K., & Göttgens, B. (2017). Reconstructing blood stem cell regulatory network models from single-cell molecular profiles. *Proceedings of the National Academy of Sciences, USA*, *114*, 5822–5829. https://doi.org/10.1073/pnas.1610609114.

Hanley, J. A., & McNeil, B. J. (1982). The meaning and use of the area under a receiver operating characteristic (ROC) curve. *Radiology*, *143*, 29–36. https://doi.org/10.1148/radiology.143.1.7063747.

Haury, A.-C., Mordelet, F., Vera-Licona, P., & Vert, J.-P. (2012). TIGRESS: Trustful inference of gene regulation using stability selection. *BMC Systems Biology*, *6*, 1–17. https://doi.org/10.1186/1752-0509-6-145.

Huynh-Thu, V. A., Irrthum, A., Wehenkel, L., & Geurts, P. (2010). Inferring regulatory networks from expression data using tree-based methods. *PLoS One*, *5*, e12776. https://doi.org/10.1371/journal.pone.0012776.

James, G., Witten, D., Hastie, T., & Tibshirani, R. (2013). *An introduction to statistical learning* volume 112. Springer. https://link.springer.com/content/pdf/10.1007/978-1-0716-1418-1.pdf.

Li, L., & Liu, Z.-P. (2020). Biomarker discovery for predicting spontaneous preterm birth from gene expression data by regularized logistic regression. *Computational and Structural Biotechnology Journal*, *18*, 3434–3446. https://doi.org/10.1016/j.csbj.2020.10.028.

Liang, Y., Liu, C., Luan, X.-Z., Leung, K.-S., Chan, T.-M., Xu, Z.-B., & Zhang, H. (2013). Sparse logistic regression with a $L_{1/2}$ penalty for gene selection in cancer classification. *BMC Bioinformatics*, *14*, 1–12. https://doi.org/10.1186/1471-2105-14-198.

Liu, X., Wang, Y., Shi, N., Ji, Z., & He, S. (2021). GAPORE: Boolean network inference using a genetic algorithm with novel polynomial representation and encoding scheme. *Knowledge-Based Systems*, *228*, 107277. https://doi.org/10.1016/j.knosys.2021.107277.

Margolin, A. A., Nemenman, I., Basso, K., Wiggins, C., Stolovitzky, G., Favera, R. D., & Califano, A. (2006). ARACNE: An algorithm for the reconstruction of gene regulatory networks in a mammalian cellular context. *BMC Bioinformatics*, *7*, 1–15. https://doi.org/10.1186/1471-2105-7-S1-S7.

Matsumoto, H., Kiryu, H., Furusawa, C., Ko, M. S., Ko, S. B., Gouda, N., Hayashi, T., & Nikaido, I. (2017). SCODE: An efficient regulatory network inference algorithm from single-cell RNA-Seq during differentiation. *Bioinformatics*, *33*, 2314–2321. https://doi.org/10.1093/bioinformatics/btx194.

Müssel, C., Schmid, F., Blätte, T. J., Hopfensitz, M., Lausser, L., & Kestler, H. A. (2016). BiTrinA-multiscale binarization and trinarization with quality analysis. *Bioinformatics*, *32*, 465–468. https://doi.org/10.1093/bioinformatics/btv591.

Papili Gao, N., Ud-Dean, S. M., Gandrillon, O., & Gunawan, R. (2018). SINCERITIES: Inferring gene regulatory networks from time-stamped single cell transcriptional expression profiles. *Bioinformatics*, *34*, 258–266. https://doi.org/10.1093/bioinformatics/btx575.

Qiu, P. (2020). Embracing the dropouts in single-cell RNA-seq analysis. *Nature Communications*, *11*, 1–9. https://doi.org/10.1038/s41467-020-14976-9.

Schaffter, T., Marbach, D., & Floreano, D. (2011). GeneNetWeaver: In silico benchmark generation and performance profiling of network inference methods. *Bioinformatics*, *27*, 2263–2270. https://doi.org/10.1093/bioinformatics/btr373.

Schwab, J. D., Ikonomi, N., Werle, S. D., Weidner, F. M., Geiger, H., & Kestler, H. A. (2021). Reconstructing Boolean network ensembles from single-cell data for unraveling dynamics in the aging of human hematopoietic stem cells. *Computational and Structural Biotechnology Journal*, *19*, 5321–5332. https://doi.org/10.1016/j.csbj.2021.09.012.

Seçilmiş, D., Hillerton, T., Tjärnberg, A., Nelander, S., Nordling, T. E., & Sonnhammer, E. L. (2022). Knowledge of the perturbation design is essential for accurate gene regulatory network inference. *Scientific Reports*, *12*, 1–12. https://doi.org/10.1038/s41598-022-19005-x.

Treutlein, B., Lee, Q. Y., Camp, J. G., Mall, M., Koh, W., Shariati, S. A. M., Sim, S., Neff, N. F., Skotheim, J. M., Wernig, M. et al. (2016). Dissecting direct reprogramming from fibroblast to neuron using single-cell RNA-seq. *Nature*, *534*, 391–395. https://doi.org/10.1038/nature18323.

Wang, J., Huang, Q., Liu, Z.-P., Wang, Y., Wu, L.-Y., Chen, L., & Zhang, X.-S. (2011). NOA: A novel Network Ontology Analysis method. *Nucleic Acids Research*, *39*, e87–e87. https://doi.org/10.1093/nar/gkr251.

Zhang, Y., Chang, X., & Liu, X. (2021). Inference of gene regulatory networks using pseudo-time series data. *Bioinformatics*, *37*, 2423–2431. https://doi.org/10.1093/bioinformatics/btab099.
